# Feline-Derived *Lactiplantibacillus plantarum*–Aloe Vera Polysaccharide Hydrogel as a Topical Control Strategy for *Staphylococcus pseudintermedius*-Infected Wounds

**DOI:** 10.3390/vetsci13101126

**Published:** 2026-10-21

**Authors:** Qianqian Chen, Hao Dong, Weiwei Wang, Huakai Wang, Jiahang Yao, Mengyao Ma, Shiqiang Zhu, Longjiao Zhu, Zaihui Du, Zhaofei Xia, Wei Xiong

**Affiliations:** 1 Food Laboratory of Zhongyuan, Luohe 462300, China; 2020920269@stu.haut.edu.cn (Q.C.); donghao980623@stu.haut.edu.cn (H.D.);; 2 Department of Nutrition and Health, China Agricultural University, Beijing 100083, China; 3 Institute of Food and Nutrition Development, Ministry of Agriculture and Rural Affairs, Beijing 100083, China; 4 College of Veterinary Medicine, China Agricultural University, Beijing 100094, China

**Keywords:** *Staphylococcus pseudintermedius*, *Lactiplantibacillus plantarum*, companion animals, veterinary infectious disease, probiotic therapy, biofilm, wound microbiota, antimicrobial stewardship, One Health

## Abstract

*Staphylococcus pseudintermedius* (SP) is an important opportunistic pathogen in companion animals, and the increasing prevalence of antimicrobial-resistant strains highlights the need for alternative approaches to local infection control. This study evaluated a topical hydrogel containing feline-derived *Lactiplantibacillus plantarum* ZYpet-014 and Aloe vera polysaccharides (AVEs) for the treatment of SP-infected wounds. AVEs promoted LP growth and organic-acid production and improved short-term LP viability within the carbomer hydrogel. The LP+AVE-hydrogel inhibited planktonic SP growth and biofilm formation in vitro and caused pronounced morphological disruption of SP cells. Therapeutic efficacy was further evaluated using a full-thickness murine wound model challenged with SP. LP+AVE treatment accelerated wound closure, reaching 95.75% by day 14, while qualitative histological observations suggested improved tissue organization and differences in collagen-associated staining patterns. Treatment was also associated with reduced TNF-α, IL-1β, and IL-6 levels and increased IL-4, IL-10, TGF-β1, and serum VEGF concentrations. In wound tissues, LP+AVE treatment decreased CD80 and increased CD206 immunoreactivity, consistent with a shift in macrophage-associated responses toward a reparative profile. Moreover, 16S rRNA gene sequencing showed reduced relative abundance of Staphylococcus and increased relative abundance of Lactobacillus in treated wounds. Together, these findings demonstrate antibacterial and antibiofilm activities against SP in vitro, accompanied by improved wound healing, modulation of inflammatory responses, and changes in wound microbial composition in vivo, although direct reduction in SP burden in wound tissue remains to be established.

## Simple Summary

*Staphylococcus pseudintermedius* is an important cause of skin and wound infections in companion animals, and antimicrobial-resistant strains are increasingly difficult to manage. We developed a topical hydrogel containing *Lactiplantibacillus plantarum* isolated from a healthy cat together with Aloe vera polysaccharides. The formulation inhibited *Staphylococcus pseudintermedius* growth and biofilm formation in vitro. In mice with infected full-thickness skin wounds, treatment accelerated wound closure, reduced inflammatory signals, increased serum VEGF levels, and was associated with qualitative histological changes in tissue organization and alterations in wound microbial composition. These findings provide proof of concept that a host-associated probiotic hydrogel may serve as a non-antibiotic local strategy for veterinary wound infection. Because the challenge strain was not characterized for antimicrobial resistance and efficacy was evaluated in mice, further studies using resistant veterinary isolates and naturally occurring canine or feline infections are required.

## 1. Introduction

*Staphylococcus pseudintermedius* (SP) is a major opportunistic bacterial pathogen of companion animals and is particularly associated with canine skin and soft-tissue infections. Although SP can form part of the normal canine skin and mucosal microbiota, disruption of cutaneous homeostasis or host defenses can facilitate infection, including pyoderma, wound infection, otitis, and postoperative infection [1,2]. Management has become increasingly challenging because methicillin-resistant S. pseudintermedius (MRSP) frequently displays resistance to multiple antimicrobial classes, restricting therapeutic options in veterinary practice [3,4,5]. Biofilm formation represents an additional concern, as biofilm-producing SP isolates have been recovered from canine pyoderma and skin lesions and may contribute to bacterial persistence and reduced antimicrobial susceptibility [6,7,8,9]. SP is also increasingly recognized as an emerging zoonotic pathogen, with human colonization and infection reported particularly among individuals in close contact with companion animals and evidence supporting probable transmission between dogs and their owners [10,11,12]. These features highlight the need for alternative local strategies capable of controlling SP infection without relying exclusively on conventional antimicrobials.

Probiotic-based approaches have attracted increasing attention as biological strategies for wound infection because selected strains and their metabolites can inhibit pathogen growth, interfere with biofilm development, and modulate inflammatory responses [13,14,15,16]. Among these microorganisms, *Lactiplantibacillus plantarum* (LP) has shown particular promise in experimental wound models. LP or LP-derived preparations have been reported to suppress staphylococcal growth and biofilms, reduce bacterial burden, and promote wound repair in vitro and in vivo [17,18,19]. More recently, viable probiotics have been incorporated into living hydrogel systems to improve local retention and preserve biological activity at wound surfaces [20,21,22]. Importantly, probiotic effects are strain dependent, creating interest in host-associated microorganisms as potential resources for companion-animal applications. Lactic acid bacteria isolated from feline feces have demonstrated probiotic-associated properties in vitro, supporting the feline microbiota as a potential source of functional strains [23]. However, host origin alone does not establish superior therapeutic performance or specific adaptation to cutaneous environments. Whether a feline-derived LP strain can remain functionally active in a topical formulation and suppress SP-associated wound infection therefore remains to be determined.

Aloe vera contains biologically active polysaccharides, particularly acetylated mannans such as acemannan, that may complement probiotic-based wound treatment. Acemannan and other Aloe-derived polysaccharides have been associated with fibroblast proliferation, keratinocyte migration and differentiation, collagen synthesis, inflammatory regulation, and tissue repair [24,25,26]. Aloe-containing wound materials have also shown antibacterial, anti-inflammatory, and repair-associated effects in experimental systems [27,28,29,30]. In addition, Aloe polysaccharides can exhibit prebiotic-associated properties and influence microbial growth and metabolic output [31,32]. These observations provide a rationale for evaluating AVE together with a living probiotic, with the possibility that AVE may support LP growth or metabolic activity while also contributing independently to the wound-repair environment. Nevertheless, such an interaction should be demonstrated experimentally rather than assumed to represent probiotic–prebiotic synergy.

Hydrogels provide hydrated three-dimensional matrices capable of maintaining close contact with wound surfaces and incorporating antimicrobial or biologically active components [33]. They have therefore been increasingly investigated as local delivery platforms for probiotics and probiotic-derived products [34]. Living probiotic hydrogels can preserve bacterial viability, sustain local metabolic activity, and provide antimicrobial effects at infected wound sites [20,21,22]. In the present study, the carbomer hydrogel was used primarily as a practical topical carrier rather than developed as a novel biomaterial. This distinction is important because the therapeutic concept centers on the biological activity of LP and AVE within the infected wound microenvironment rather than on a new polymeric mechanism.

Successful resolution of an infected wound requires more than the suppression of planktonic bacterial growth. Persistent infection and biofilm formation can prolong inflammatory responses and interfere with re-epithelialization, vascularization, extracellular-matrix deposition, and tissue remodeling [35]. In parallel, wound healing is increasingly understood to involve interactions among invading pathogens, resident microbial communities, and host immunity [36,37,38,39,40]. A topical probiotic strategy capable of simultaneously restricting a dominant pathogen and modifying the local inflammatory and microbial environment may therefore provide advantages beyond conventional direct antimicrobial activity.

Based on these considerations, we hypothesized that combining feline-derived LP ZYpet-014 with AVE in a carbomer hydrogel could provide a multifunctional, non-antibiotic local strategy against SP-associated wound infection. We first evaluated the effects of AVE on LP growth and organic-acid production, LP viability following incorporation into the hydrogel, basic hydrogel properties and hemocompatibility, and antibacterial and antibiofilm activity against SP in vitro. Therapeutic effects were subsequently assessed in an SP-challenged full-thickness murine wound model through measurements of wound closure, histological repair, inflammatory mediators, CD80/CD206-associated immunoreactivity, and wound microbial composition. The study was designed to determine whether local delivery of a companion-animal-derived probiotic could simultaneously suppress SP-associated pathogenic features and promote an infected-wound environment more compatible with tissue repair.

## 2. Materials and Methods

### 2.1. Bacterial Strains and Materials

The feline-derived *Lactiplantibacillus plantarum* (LP) strain ZYpet-014 used in this study was isolated from the feces of a healthy domestic cat. Its taxonomic identification was supported by 16S rRNA gene sequencing and phylogenetic analysis, and the strain was deposited in the China General Microbiological Culture Collection Center (CGMCC; accession No. 32010). Additional strain characterization was performed using 16S rRNA gene-based phylogenetic analysis, disk-diffusion antimicrobial susceptibility screening, and targeted PCR assays for selected virulence-associated and antibiotic-resistance genes. The corresponding methods and results are provided in Supplementary Method S1, Supplementary Figure S2, and Supplementary Tables S1 and S2. *Staphylococcus pseudintermedius* (SP; ATCC 49444) was obtained from the Shanghai Microbiological Culture Collection Center (Shanghai, China). A commercial Aloe vera-derived preparation (AVE; supplier-declared purity ≥60%, Cat. No. S27799) was purchased from Shanghai Yuanye Bio-Technology Co., Ltd. (Shanghai, China). The detailed polysaccharide composition and remaining constituents of AVE were not independently characterized. Carbomer 934 was obtained from Shanghai McLean Biochemical Technology Co., Ltd. (Shanghai, China). Primary antibodies against TNF-α (rabbit polyclonal, Servicebio, GB115702), IL-10 (rabbit polyclonal, Servicebio, GB11534), CD206 (rabbit polyclonal, Servicebio, GB113497), and CD80 (rabbit polyclonal, Servicebio, GB114055) were used for immunofluorescence staining.

### 2.2. Effect of AVE on the Growth of LP

To determine whether AVE affected the growth of LP, MRS broth was supplemented with AVE to final concentrations of 0, 2.5, 5, 10, 20, 40, or 80 mg/mL. Each medium was inoculated with 1% (*v*/*v*) overnight LP culture and incubated at 37 °C with shaking at 220 rpm for 12 h. Bacterial growth was evaluated by measuring the optical density at 600 nm (OD600). To eliminate potential interference from the intrinsic absorbance or turbidity of AVE, uninoculated MRS media containing the corresponding AVE concentrations (0, 2.5, 5, 10, 20, 40, and 80 mg/mL) were used as concentration-matched blanks. The OD600 values of the LP cultures were corrected by subtracting the absorbance of the corresponding uninoculated medium at each AVE concentration. The background-corrected OD600 was calculated as follows:

OD600,corrected = OD600,sample − OD600,blank

where OD600,sample represents the absorbance of the LP-inoculated culture and OD600,blank represents the absorbance of the uninoculated medium containing the same AVE concentration. The corrected OD600 values were used for comparisons of bacterial growth among treatment groups.

### 2.3. Determination of Organic-Acid Production

LP was cultured in 5 mL of MRS broth with or without 5 mg/mL AVE at 37 °C for 48 h, and culture supernatants were collected for organic-acid analysis. Samples were extracted using methanol/chloroform (7:3, *v*/*v*), incubated on ice for 30 min, mixed with ultrapure water, and centrifuged at 12,000 rpm for 10 min at 4 °C. The collected extracts were derivatized with 0.1 M 3-nitrophenylhydrazine (3NPH) and 0.1 M 1-(3-dimethylaminopropyl)-3-ethylcarbodiimide (EDC) at 40 °C for 30 min. Organic acids were analyzed using a Vanquish ultra-high-performance liquid chromatography (UHPLC) system coupled to a Q Exactive high-resolution mass spectrometer (Thermo Fisher Scientific, Waltham, MA, USA). Chromatographic separation was performed on a Waters ACQUITY UPLC BEH C18 column (50 × 2.1 mm, 1.8 μm; Waters Corporation, Milford, MA, USA) using water (A) and acetonitrile (B), with the mobile-phase additives specified according to the original analytical protocol. The gradient increased from 10% to 90% B between 2 and 12 min. The flow rate was 0.35 mL/min, the column temperature was 40 °C, and the injection volume was 2 μL. Mass spectrometric detection was performed in negative electrospray ionization mode. Analytes were quantified using external calibration curves prepared from authentic reference standards over a reported concentration range of 1–20,000 ng/mL, and the data were processed using TraceFinder 4.1 software. Results were expressed as endpoint organic-acid concentrations in culture samples rather than biomass-normalized production rates.

### 2.4. Preparation of LP-AVE Composite Carbomer Hydrogels

All hydrogel formulations were prepared aseptically in a laminar-flow clean bench using sterile distilled water. Briefly, Carbomer 934 (150 mg) was dispersed in 20 mL of sterile distilled water and allowed to hydrate completely. Triethanolamine (100 mg) and glycerol (500 mg) were then added under continuous mixing to obtain the blank carbomer hydrogel (CON-hydrogel). The hydrogel matrix was not subjected to heat sterilization. For LP-hydrogel preparation, LP cells were harvested from overnight MRS cultures, washed with sterile physiological saline, and aseptically incorporated into the hydrogel matrix to achieve a final viable concentration of approximately 1 × 10^8^ CFU/mL. For the LP+AVE-hydrogel, AVE solution was filter-sterilized through a 0.22 μm membrane before incorporation at a final concentration of 5 mg/mL. The final volume of each formulation was approximately 20.5–21 mL, and the initial pH was adjusted to 6.5–7.0. Each formulation (CON-hydrogel, LP-hydrogel, and LP+AVE-hydrogel) was independently prepared in three separate batches (*n* = 3 batches per formulation). The viable LP concentration in freshly prepared LP-containing hydrogels was verified by plate counting.

### 2.5. Fluorescence-Based Assessment of LP Membrane Integrity After Short-Term Storage

The membrane-integrity-associated fluorescence characteristics of LP following incorporation into the hydrogel were examined using SYTO 9/propidium iodide (PI) dual staining, adapted from Xu et al. (2024) [21]. Equivalent LP suspensions without hydrogel (LP-Free), LP-hydrogel, and LP+AVE-hydrogel were stored at 4 °C for 48 h. Samples were stained with 5 μM SYTO 9 and 2 μg/mL PI and incubated at 37 °C for 15 min in the dark. A 10 μL aliquot of each stained sample was examined using a laser-scanning confocal microscope (FV1200, Olympus Corporation, Tokyo, Japan) at 200× magnification. SYTO 9-associated green fluorescence and PI-associated red fluorescence were acquired under identical imaging settings and analyzed using ImageJ software (version 1.53t; National Institutes of Health, Bethesda, MD, USA). The relative SYTO 9 fluorescence proportion was calculated as follows:

Relative SYTO 9 fluorescence proportion (%) = [Igreen/(Igreen + Ired)] × 100%.

where Igreen and Ired represent the background-corrected fluorescence intensities of the green and red channels, respectively. Measurements from multiple microscopic fields of the same sample were averaged before statistical analysis. This fluorescence-derived parameter was used to compare membrane-integrity-associated staining characteristics and was not considered equivalent to the percentage of culturable viable bacteria. Parallel CFU measurements after 48 h of storage and independent validation of dye- and matrix-related fluorescence interference were not performed.

### 2.6. Morphological Characterization of Hydrogels

The internal morphology of CON-hydrogel, LP-hydrogel, and LP+AVE-hydrogel was examined by scanning electron microscopy (SEM; Sigma 30, Zeiss, Oberkochen, Germany). Hydrogel samples were freeze-dried and fractured in liquid nitrogen. The exposed cross-sectional surfaces were mounted on SEM stubs and sputter-coated with gold before imaging. Samples were observed at magnifications of 300× and 2000× to assess the overall porous architecture of the hydrogel matrix and the distribution and morphology of incorporated LP cells. The SEM observations were used as morphological characterization and were not interpreted as quantitative measurements of pore size.

### 2.7. Swelling Behavior of Hydrogels

The swelling behaviors of CON-hydrogel, LP-hydrogel, and LP+AVE-hydrogel were evaluated in 0.01 M PBS (pH 7.4). Freeze-dried hydrogel samples were first weighed to obtain their initial dry mass (m0). Samples were then immersed in PBS, and swelling was determined at 0, 100, 200, 300, and 400 min. At each time point, samples were removed from PBS, and excess surface liquid was gently blotted using filter paper before weighing (mt). The samples were subsequently returned to PBS for continued incubation. The swelling ratio was calculated as:

Swelling ratio (%) = [(mt − m_0_)/m_0_] × 100%.

where m0 is the initial dry weight and mt is the weight of the hydrated hydrogel at the specified time point.

### 2.8. pH Changes in the Hydrogel Microenvironment

To evaluate hydrogel-associated changes in the surrounding pH, 1.0 g of CON-hydrogel, LP-hydrogel, or LP+AVE-hydrogel was immersed in 4 mL PBS (initial pH 7.4) at 25 °C. The pH of the surrounding medium was measured at 0, 1, 2, 3, 4, 5, and 6 h using a calibrated pH meter. Measurements were performed under identical conditions for all formulations. Changes in pH were used to evaluate acidification of the hydrogel-associated microenvironment resulting from metabolic activity of incorporated LP.

### 2.9. Hemolytic Activity and Hemocompatibility

The hemolytic activity of LP was first qualitatively screened on blood agar. LP was inoculated onto blood agar plates and incubated at 37 °C, and the presence or absence of a visible hemolytic halo was assessed relative to the positive hemolytic control used in the assay. Quantitative hemolysis of the hydrogel formulations was subsequently evaluated using a method adapted from Zheng et al. [41]. Fresh anticoagulated rabbit blood was diluted with sterile physiological saline and centrifuged at 1400 rpm for 4 min. The erythrocyte fraction was collected and resuspended in physiological saline for subsequent testing. The experimental groups consisted of CON-hydrogel, LP-hydrogel, LP+AVE-hydrogel, PBS as the negative control, and 0.2% (*v*/*v*) Triton X-100 as the positive control. For each experimental formulation, 0.4 mL hydrogel was mixed with 0.2 mL erythrocyte suspension and incubated at 37 °C for 2 h. The mixtures were subsequently centrifuged at 2500 rpm for 4 min, and 200 μL of the resulting supernatant was transferred to a 96-well plate. Absorbance was measured at 545 nm using a microplate reader. The hemolysis rate was calculated as:

Hemolysis rate (%) = [(OD_s_ − OD_n_)/(OD_p_ − OD_n_)] × 100

where OD_s_ represents the absorbance of the experimental group, OD_n_ represents the absorbance of the negative control, and OD_p_ represents the Triton X-100 positive control.

### 2.10. Antibacterial Activity Against Staphylococcus pseudintermedius

The antibacterial activity of the hydrogel formulations against SP was evaluated using agar well-diffusion and viable-counting assays. For the agar well-diffusion assay, LB agar was inoculated with 1% SP suspension adjusted to approximately 1 × 10^7^ CFU/mL and poured into sterile Petri dishes. After solidification, 10 mm diameter wells were punched into the agar using a sterile borer, and 200 μL of each hydrogel formulation was added to the corresponding well. Plates were incubated at 37 °C for 24 h. The total diameter of the clear zone was measured, and the net inhibition-zone diameter was calculated as:

Dnet = (Dtotal– Dwell); where Dwell = 10 mm.

For the SP viable-counting assay, 0.5 mL of each hydrogel formulation was mixed with 3 mL of SP suspension (approximately 1 × 10^7^ CFU/mL) and incubated at 37 °C with shaking at 220 rpm for 24 h. PBS served as the untreated control. To validate the suitability of LB agar for selective SP enumeration in the LP–SP co-culture system, pure LP, pure SP, and mixed LP+SP suspensions were separately inoculated onto LB agar plates. Under the tested plating conditions, pure LP did not produce visible colonies, whereas SP formed colonies normally, and colony growth was observed from the mixed inoculum. These observations supported the use of LB agar for preferential recovery and enumeration of SP in the present two-strain system, with negligible detectable interference from LP. Following hydrogel treatment, bacterial suspensions were serially diluted in sterile physiological saline, and 100 μL aliquots of the appropriate dilutions were spread onto LB agar plates. Plates containing 30–300 colonies were selected for enumeration. Viable counts were calculated as CFU/mL = colony count × dilution factor × 10 and expressed as log_10_ CFU/mL. Each experiment was performed in triplicate. The selectivity of this approach was established for the specific LP and SP strains under the tested culture conditions and should not be generalized to other bacterial strains or culture conditions.

### 2.11. Crystal-Violet Assessment of Attached Biomass

The effects of the hydrogel formulations on attached biomass were evaluated using a crystal-violet staining assay. SP was cultured to the logarithmic growth phase and adjusted to approximately 1 × 10^7^ CFU/mL. Each hydrogel formulation (0.5 mL) was mixed with 2 mL of SP suspension in 24-well plates and incubated at 37 °C for 24 h. Following incubation, planktonic cells and culture medium were removed, and the wells were washed two to three times with sterile PBS. After drying at 37 °C for 10 min, the attached material was stained with 0.1% crystal violet for 15 min. Excess dye was removed by washing, and the retained crystal violet was solubilized with 30% acetic acid. The resulting solution was diluted fivefold, and absorbance was measured at 595 nm. The relative reduction in crystal-violet-stained biomass was calculated as [(Acontrol − Atreatment)/Acontrol] × 100%, where Acontrol and Atreatment represent the absorbance values of the control and hydrogel-treated wells, respectively. As crystal violet measures total attached biomass, the assay does not distinguish SP from LP or separate biofilm-specific inhibition from general growth suppression. Potential contributions from LP-associated biomass and the hydrogel matrix were therefore considered when interpreting the results.

### 2.12. SEM Observation Following Exposure to Hydrogel-Conditioned Media

To examine bacterial morphological changes following exposure to hydrogel-conditioned media, the different hydrogel formulations were incubated in MRS broth at 37 °C for 24 h at a hydrogel-to-medium ratio of 1:20. The conditioned media were collected by centrifugation and subsequently mixed with SP suspensions (approximately 1 × 10^7^ CFU/mL) at a conditioned-medium-to-SP-suspension volume ratio of 1:10 (*v*/*v*). The mixtures were incubated at 37 °C with shaking at 200 rpm for 24 h. Bacterial cells were collected by centrifugation and fixed in 2.5% glutaraldehyde at 4 °C in the dark for 12 h. Fixed samples were dehydrated through a graded ethanol series of 30%, 50%, 70%, 80%, 90%, and 95% for 15 min at each concentration, followed by two treatments with absolute ethanol for 20 min each. Samples were then treated with a 1:1 mixture of ethanol and isoamyl acetate for 30 min, followed by pure isoamyl acetate for 1 h. After critical-point drying, samples were sputter-coated with gold and examined by SEM. Because the conditioned media were collected by centrifugation without documented verification of complete bacterial removal, the possibility of residual LP could not be excluded. Therefore, the SEM observations were interpreted as qualitative morphological findings rather than definitive evidence of SP-specific structural damage caused exclusively by cell-free metabolites.

### 2.13. In Vivo Evaluation in an SP-Infected Full-Thickness Wound Model

#### 2.13.1. Animals and Experimental Design

All animal procedures were reviewed and approved by the Animal Care and Use Committee of China Agricultural University (Approval No. AW42405202-1-09). Male specific pathogen-free Kunming mice were purchased from Beijing SPF Biotechnology Co., Ltd. (Beijing, China). Animals were housed in individually ventilated cages under controlled environmental conditions (20–25 °C and 30–70% relative humidity) with ad libitum access to standard feed and water. Following a 7-day acclimation period, mice were randomly allocated into five groups (*n* = 12 per group): non-infected saline control (CON); SP-infected untreated group (Model); SP-infected + blank hydrogel (CON-hydrogel); SP-infected + LP-hydrogel (LP-hydrogel); SP-infected + LP+AVE-hydrogel (LP+AVE-hydrogel).

The sample size allowed six mice per group to undergo terminal sampling at each of the two predefined time points, days 7 and 14. For wound creation, mice were deeply anesthetized by intraperitoneal administration of 4% chloral hydrate at 400 mg/kg. Adequate anesthesia was confirmed by absence of the pedal-withdrawal reflex and lack of response to surgical stimulation. Dorsal hair was removed, and the surgical area was disinfected with iodophor. A single circular full-thickness excisional wound, approximately 10 mm in diameter, was created on the dorsal skin of each mouse under general anesthesia. No additional perioperative or postoperative analgesic agents were administered. Animals were monitored during recovery from anesthesia and throughout the experimental period for respiratory changes, abnormal posture, reduced activity, and other signs of distress. Wounds in the CON group were not inoculated with bacteria. Each wound in the four infected groups received 30 μL SP suspension at 1 × 10^7^ CFU/mL. Infection was allowed to establish for 48 h. The time point immediately before initiation of treatment, 48 h after bacterial inoculation, was defined as day 0. Mice in the CON-hydrogel, LP-hydrogel, and LP+AVE-hydrogel groups received 1 mL of the corresponding hydrogel per mouse per day, with the hydrogel applied topically to the wound sites. Mice in the CON and Model groups received an equivalent volume of sterile saline on the same schedule. Wounds were re-dressed daily. Animals were monitored throughout the experiment for respiration, recovery from anesthesia, abnormal posture, reduced activity, and other adverse reactions.

#### 2.13.2. Wound Measurement and Sample Collection

Wounds were photographed under standardized imaging conditions on days 0, 3, 7, 10, and 14. Longitudinal wound-closure measurements were performed in the same three mice per treatment group (*n* = 3 biological replicates) throughout the observation period. Each mouse had a single dorsal wound, and wound areas were quantified using ImageJ software by an investigator blinded to group allocation. The individual mouse was considered the biological experimental unit. Wound closure was calculated as follows: wound closure (%) = [(A_0_ − A_t_)/A_0_] × 100, where A_0_ represents the wound area immediately before initiation of treatment and A_t_ represents the wound area at the indicated time point. The same three mice were monitored longitudinally until day 14, ensuring a consistent biological sample size across all wound-closure measurements.

On days 7 and 14, six mice from each group were randomly selected for terminal sample collection. Before euthanasia, mice were deeply anesthetized by intraperitoneal administration of 10% chloral hydrate at 300 mg/kg. Deep anesthesia was confirmed by loss of the pedal-withdrawal reflex and absence of response to external stimulation. Whole blood was collected under deep anesthesia and allowed to clot before serum preparation. Animals were subsequently euthanized by cervical dislocation while under deep anesthesia, and death was confirmed by cessation of respiration and heartbeat. Full-thickness wound tissues including the wound bed and adjacent margin were collected for histological staining, immunofluorescence, and microbiota analysis. Because six mice per group underwent terminal collection on day 7, wound measurements on days 10 and 14 were obtained from the six animals per group remaining for day-14 terminal sampling.

#### 2.13.3. Histological Examination of Wound Tissue

Wound tissues collected on days 7 and 14 were fixed, routinely processed, embedded in paraffin, and sectioned for histological examination. Hematoxylin and eosin (H&E) staining was performed to assess epidermal continuity, granulation tissue organization, and inflammatory-cell infiltration. Masson’s trichrome and Sirius Red staining were used to examine collagen-associated staining patterns and tissue organization. Representative microscopic fields were selected from anatomically relevant regions encompassing the wound bed and adjacent wound margins, with comparable tissue regions prioritized across experimental groups. Images were acquired under consistent imaging conditions and evaluated qualitatively. No quantitative histomorphometric measurements or formal histological scoring data were generated; therefore, these observations were interpreted as descriptive evidence of wound-tissue organization rather than quantitative differences in re-epithelialization or collagen remodeling.

#### 2.13.4. Immunofluorescence Analysis of Wound Tissue

Paraffin-embedded wound sections collected on days 7 and 14 were subjected to immunofluorescence staining. Sections were deparaffinized and rehydrated, followed by heat-induced antigen retrieval using the appropriate antigen-retrieval buffer. After cooling to room temperature, sections were washed three times with PBS (pH 7.4) for 5 min each and blocked with 3% bovine serum albumin (BSA) for 30 min at room temperature. Sections were incubated overnight at 4 °C with rabbit polyclonal primary antibodies against TNF-α (Servicebio, GB115702; 1:500), IL-10 (Servicebio, GB11534; 1:500), CD206 (Servicebio, GB113497; 1:500), or CD80 (Servicebio, GB114055; 1:500). After three washes with PBS, sections were incubated with CY3-conjugated goat anti-rabbit IgG (Servicebio, GB21303; 1:300) for 1 h at room temperature in the dark. Nuclei were counterstained with DAPI (Servicebio, G1012) for 10 min at room temperature. Tissue autofluorescence was quenched using an autofluorescence-quenching reagent (Servicebio, G1221), and sections were mounted with anti-fade mounting medium (Servicebio, G1401). Fluorescence images were acquired using a Nikon ECLIPSE C1 fluorescence microscope under identical acquisition settings for each marker. For each animal, five randomly selected, non-overlapping microscopic fields were analyzed at 200× magnification (20× objective) within the wound region, with an individual field area of approximately 0.25 mm^2^ (500 × 500 μm). Regions of interest (ROIs) were delineated to include the wound bed and granulation tissue, while excluding adjacent intact skin, tissue folds, and imaging artifacts. Fluorescence signals were quantified using ImageJ software (National Institutes of Health, Bethesda, MD, USA). Background fluorescence was corrected using the rolling-ball subtraction method with a radius of 50 pixels. Positive staining was identified using Otsu’s automatic thresholding method, and the positively stained area was expressed as a percentage of the total ROI area. The same image-processing procedures and thresholding algorithm were applied consistently to all images stained for the same marker. Measurements from the five microscopic fields were averaged to obtain one biological value per mouse before statistical analysis. TNF-α and IL-10 staining was used to characterize local inflammatory responses, whereas CD80 and CD206 were interpreted as macrophage-associated markers rather than as definitive indicators of binary M1/M2 polarization.

#### 2.13.5. Serum Cytokine and Tissue-Repair Mediator Analysis

Serum levels of TNF-α, IL-1β, IL-6, IL-4, IL-10, VEGF, and TGF-β1 were measured using commercial ELISA kits obtained from Nanjing Jiancheng Bioengineering Institute (Nanjing, China), according to the manufacturer’s instructions. Samples and standards were processed according to the kit-specific protocols, and absorbance was measured using a microplate reader. Analyte concentrations were calculated from the corresponding standard curves and expressed in the units specified by the manufacturer.

#### 2.13.6. Wound Microbiota Profiling by 16S rRNA Gene Sequencing

Wound-tissue microbiota were analyzed on days 7 and 14. A total of 24 wound-tissue samples were collected for 16S rRNA gene sequencing, comprising four experimental groups (CON, Model, LP-hydrogel, and LP+AVE-hydrogel) and two sampling time points, with three independent biological replicates per group at each time point (n = 3). Accordingly, 12 samples were collected at each time point, yielding 24 samples in total. All 24 samples passed sample-level quality control and were retained for downstream microbiota analysis, with no samples excluded. Total microbial DNA was extracted using the E.Z.N.A. Soil DNA Kit (Omega Bio-tek, Norcross, GA, USA) according to the manufacturer’s protocol. DNA integrity was examined by electrophoresis on 1% agarose gels, and DNA concentration and purity were evaluated using a NanoDrop 2000 spectrophotometer (Thermo Scientific, Waltham, MA, USA). Samples with A_260_/A_280_ >1.8 and A_260_/A_230_ >2.0 were used for downstream analysis. The V3–V4 region of the bacterial 16S rRNA gene was amplified using primers 338F (5′-ACTCCTACGGGAGGCAGCAG-3′) and 806R (5′-GGACTACHVGGGTWTCTAAT-3′). PCR amplification was performed in triplicate for each sample, and replicate PCR products were pooled. Amplicons were purified using the AxyPrep DNA Gel Extraction Kit (Axygen Biosciences, Union City, CA, USA) and quantified using a Quantus Fluorometer (Promega, Madison, WI, USA). Sequencing libraries were prepared using the NEXTflex Rapid DNA-Seq Kit (Bioo Scientific, Austin, TX, USA) and subjected to paired-end sequencing on an Illumina NovaSeq platform. Raw 16S rRNA gene sequencing reads were demultiplexed and quality-filtered using fastp (v0.20.0), and paired-end reads were merged using FLASH (v1.2.7). High-quality sequences were clustered into operational taxonomic units (OTUs) at 97% sequence similarity using UPARSE (v7.1), with chimeric sequences identified and removed during processing. Representative sequences from each OTU were taxonomically classified using the Ribosomal Database Project (RDP) Classifier (v2.2) against the SILVA 16S rRNA gene database (Release 138), with a confidence threshold of 0.7 (70%). Taxonomic relative abundances were calculated from the assigned sequences and summarized primarily at the phylum and genus levels. Exploratory alpha-diversity analyses included the ACE, Chao1, Shannon, and Simpson indices. Beta-diversity patterns were visualized using principal coordinate analysis (PCoA) of genus-level microbial community data. These analyses were performed to provide complementary descriptive information on wound microbial diversity and community variation. Differences in predominant bacterial taxa among experimental groups were evaluated using the Kruskal–Wallis H test. Exploratory associations between wound microbial taxa and serum inflammatory or tissue-repair-related parameters were evaluated using Spearman rank-correlation analysis. Only matched wound microbiota and serum measurements obtained from the same animals on day 14 were included (*n* = 12 matched observations, comprising three mice from each of four experimental groups). Although serum measurements were available from six mice per group, only those corresponding to the three mice included in the microbiota analysis were used. Data from the four treatment groups were pooled for correlation analysis, whereas observations from different sampling time points were not combined.

### 2.14. Statistical Analysis

All data are presented as mean ± SEM. Statistical analyses of endpoints other than longitudinal wound closure were performed using GraphPad Prism 8.0.2 (GraphPad Software, San Diego, CA, USA). For comparisons among multiple groups at a single time point, one-way analysis of variance (ANOVA) followed by Tukey’s multiple-comparisons test was used, where appropriate. Longitudinal wound-closure data were reanalyzed using two-way repeated-measures ANOVA, with treatment as the between-subject factor and time as the within-subject factor, including the treatment × time interaction. The same three mice per treatment group were repeatedly measured on days 3, 7, 10, and 14, with each mouse treated as an independent biological experimental unit. Day 0 was used as the baseline for calculating wound closure and was excluded from the inferential analysis because wound closure was zero by definition. Greenhouse–Geisser correction was applied to the within-subject time effect and treatment × time interaction. Following the significant interaction, differences among treatment groups at individual time points were evaluated using Tukey’s honestly significant difference (HSD) test. The longitudinal repeated-measures analysis and corresponding post hoc comparisons were performed using Python with NumPy and SciPy (version 1.17.0). Statistical significance was set at *p* < 0.05.

## 3. Results

### 3.1. AVE Promotes LP Growth and Organic-Acid Production

**Figure 1:**
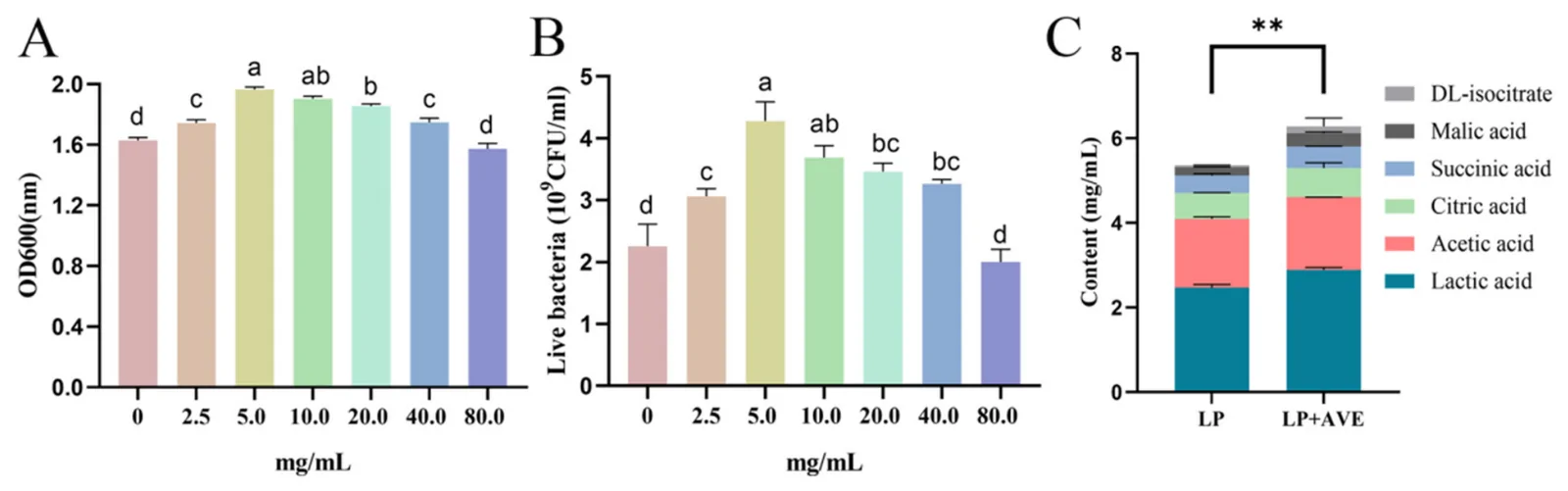
Effects of AVE on LP growth and organic-acid production. (**A**) OD_600_ of LP cultures supplemented with different concentrations of AVE. (**B**) Viable LP counts after 12 h of cultivation. (**C**) Organic-acid profiles of LP cultured in the absence or presence of 5 mg/mL AVE for 48 h. Data are presented as mean ± SEM. Different lowercase letters indicate significant differences among groups (*p* < 0.05); ** indicates *p* < 0.01.

The effects of different AVE concentrations on LP growth were first evaluated by OD_600_ measurement and viable-cell counting. After 12 h of incubation, supplementation with AVE at 2.5–40 mg/mL generally enhanced LP growth compared with the unsupplemented control (Figure 1A,B). Among the tested concentrations, 5 mg/mL AVE produced the highest viable-cell yield, reaching 4.28 × 10^9^ CFU/mL, with a corresponding OD_600_ value of 1.96. In contrast, LP growth declined at 80 mg/mL AVE. Based on these results, 5 mg/mL AVE was selected for subsequent experiments. After 48 h of cultivation, the LP+AVE group showed greater organic-acid production than LP alone (Figure 1C), with the differences particularly evident for lactic acid and acetic acid. These findings indicate that AVE at the selected concentration supported both LP proliferation and metabolic activity under the tested culture conditions.

### 3.2. Fluorescence-Based Assessment of LP After Short-Term Storage in Carbomer Hydrogels

**Figure 2:**
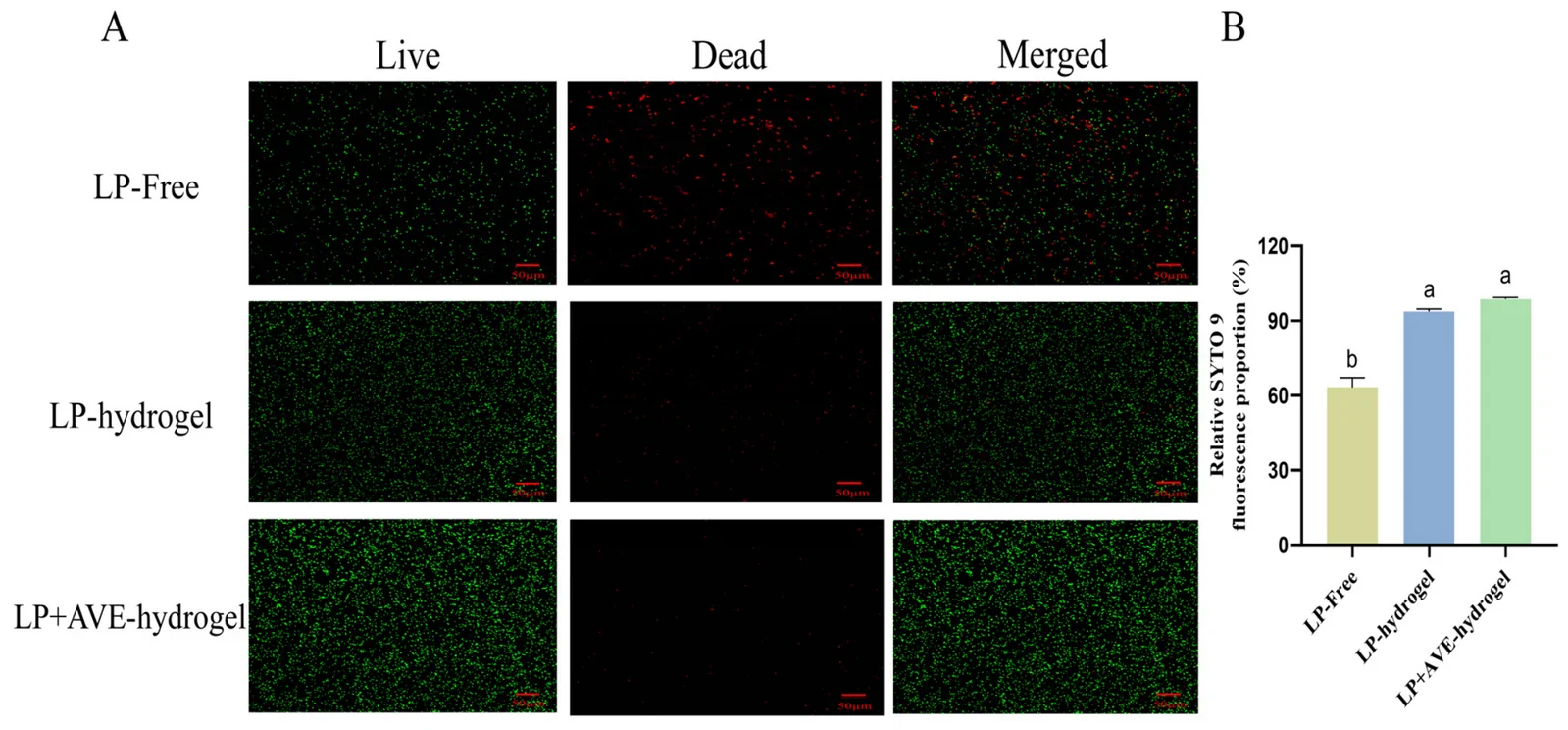
Fluorescence-based assessment of LP following short-term storage in carbomer hydrogels. (**A**) Representative SYTO 9/PI fluorescence images of LP-Free, LP-hydrogel, and LP+AVE-hydrogel after storage at 4 °C for 48 h (scale bar, 50 μm). Green and red fluorescence represent SYTO 9- and PI-associated signals, respectively. (**B**) Relative SYTO 9 fluorescence proportion calculated from background-corrected green and red fluorescence intensities. This fluorescence-derived parameter is not equivalent to culturable bacterial viability. Data are presented as mean ± SEM. Different lowercase letters indicate significant differences among groups (*p* < 0.05).

SYTO 9/PI dual staining was used to evaluate the membrane-integrity-associated fluorescence characteristics of LP after storage at 4 °C for 48 h. Representative images showed predominantly SYTO 9-associated green fluorescence in both LP-hydrogel and LP+AVE-hydrogel, with relatively limited PI-associated red fluorescence (Figure 2A). Quantitative fluorescence analysis showed that the relative SYTO 9 fluorescence proportion was significantly higher in both hydrogel formulations than in the LP-Free group (*p* < 0.05; Figure 2B). No significant difference was detected between LP-hydrogel and LP+AVE-hydrogel (*p* > 0.05). These findings indicate that LP incorporated into the hydrogel formulations exhibited more favorable membrane-integrity-associated fluorescence characteristics after short-term refrigerated storage. However, because post-storage CFU counts were not determined, these results do not establish differences in culturable bacterial survival or longer-term formulation stability.

### 3.3. Carbomer Hydrogel Supports Incorporation of LP

**Figure 3:**
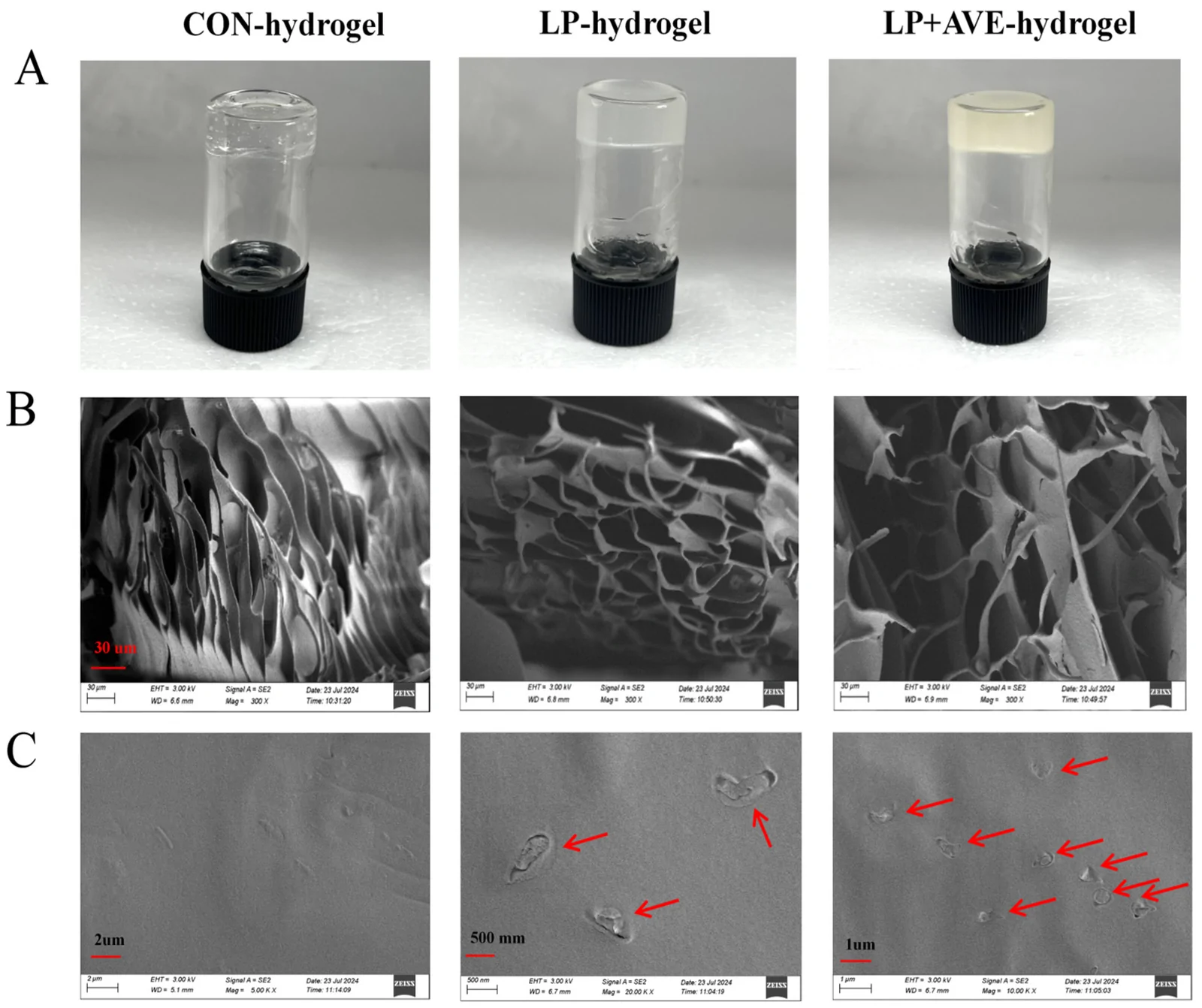
Morphological characterization of the hydrogel formulations. (**A**) Representative appearance of CON-hydrogel, LP-hydrogel, and LP+AVE-hydrogel. (**B**) SEM images showing the internal porous architecture of the hydrogels. (**C**) Higher-magnification SEM images showing rod-shaped LP cells within or on the hydrogel matrix.

All three hydrogel formulations formed stable gels in the tube-inversion test. The CON-hydrogel appeared transparent and homogeneous, whereas incorporation of LP resulted in a slightly opaque appearance in LP-hydrogel and LP+AVE-hydrogel (Figure 3A). SEM examination showed a porous three-dimensional architecture in all formulations (Figure 3B). Compared with the blank hydrogel, the LP-containing formulations exhibited a visually more open internal structure. At higher magnification, rod-shaped bacterial cells were observed within or on the surface of the hydrogel matrices in both LP-hydrogel and LP+AVE-hydrogel, confirming the successful incorporation of LP while preserving its characteristic morphology (Figure 3C).

### 3.4. LP-Containing Hydrogels Exhibit Favorable Swelling, Acidification, and Hemocompatibility

**Figure 4:**
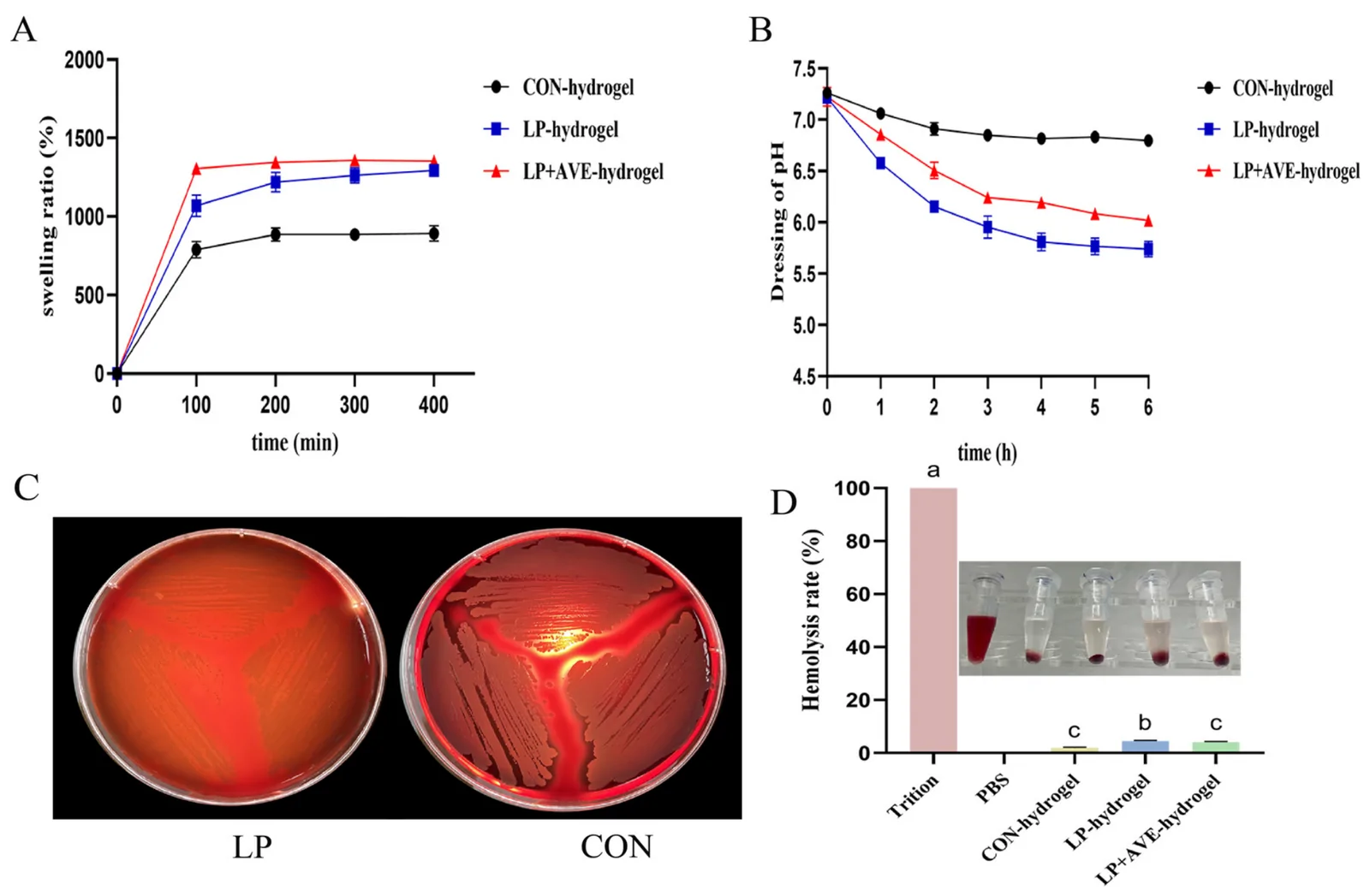
Material properties and hemocompatibility of the hydrogels. (**A**) Swelling ratio (%) of the hydrogels during immersion over time. (**B**) pH changes in the hydrogel dressings during incubation. (**C**) Blood agar hemolysis test (LP vs. positive control). (**D**) Hemolytic rates of hydrogels. The data are presented as mean ± sem, and different lowercase letters in each group show significant differences (*p* < 0.05).

All hydrogel formulations exhibited rapid water uptake during the initial stage of PBS immersion, followed by a gradual approach to equilibrium (Figure 4A). LP-hydrogel and LP+AVE-hydrogel showed higher swelling ratios than CON-hydrogel during the later stages of incubation. The pH of the surrounding medium remained relatively stable for CON-hydrogel, whereas LP-hydrogel and LP+AVE-hydrogel produced a progressive decrease in pH over 6 h (Figure 4B). The decrease was more pronounced in the LP-containing formulations, consistent with continued metabolic activity of incorporated LP. LP did not produce a visible hemolytic halo in the blood-agar assay (Figure 4C). In the erythrocyte hemolysis assay, the hemolysis rates of CON-hydrogel, LP-hydrogel, and LP+AVE-hydrogel were all below 5% and markedly lower than that of the Triton X-100 positive control (Figure 4D). Together, these findings demonstrate favorable swelling behavior, LP-associated acidification, and low hemolytic activity of the hydrogel formulations.

### 3.5. LP+AVE-Hydrogel Exerts Antibacterial and Antibiofilm Activity Against SP In Vitro

**Figure 5:**
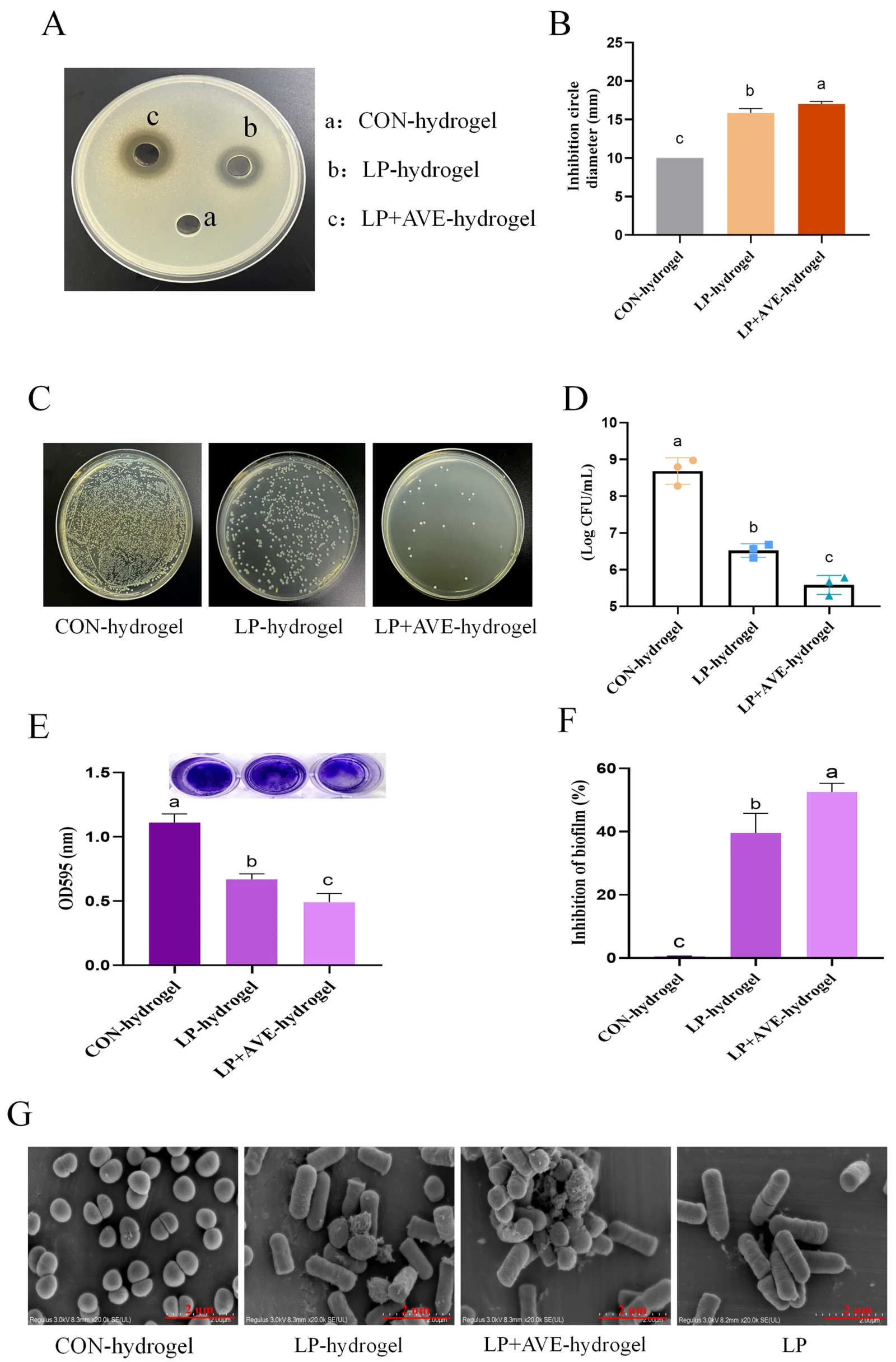
In vitro growth-inhibitory activity, bacterial colony recovery, attached-biomass measurements, and morphological observations following hydrogel treatment. (**A**) Representative agar well-diffusion assay against SP and (**B**) total inhibition-zone diameters, including the 10 mm well diameter, measured using the agar well-diffusion assay. The corresponding net inhibition-zone diameters were calculated by subtracting the well diameter (10 mm) from the total diameter. (**C**) Representative LB agar plates following incubation with the indicated hydrogel formulations and (**D**) endpoint counts of presumptive SP colonies after 24 h of treatment. (**E**) Crystal-violet staining of total attached biomass and (**F**) relative reduction in crystal-violet-stained biomass compared with the control. (**G**) Representative SEM images showing bacterial morphological characteristics after exposure to hydrogel-conditioned media, together with reference LP morphology. Crystal-violet staining was not species-specific, and SEM observations were used for qualitative morphological assessment rather than determination of bacterial viability. Data are presented as mean ± SEM. Different letters indicate significant differences among groups (*p* < 0.05).

The antibacterial effects of the hydrogel formulations against SP were evaluated using agar well-diffusion, viable-counting, crystal-violet staining, and SEM assays. CON-hydrogel produced no detectable inhibition zone, whereas both LP-containing hydrogels inhibited SP growth (Figure 5A,B). LP+AVE-hydrogel exhibited a significantly larger net inhibition-zone diameter than LP-hydrogel (7.03 vs. 5.86 mm, *p* < 0.05). Consistent with these findings, viable-counting analysis showed significantly lower recoverable SP colony counts in the LP-hydrogel and LP+AVE-hydrogel groups than in the CON-hydrogel group, with LP+AVE-hydrogel exhibiting the lowest endpoint count of approximately 5.5 log_10_CFU/mL after 24 h (Figure 5C,D). Crystal-violet staining revealed reduced attached biomass following treatment with LP-containing hydrogels (Figure 5E), with LP+AVE-hydrogel producing a significantly greater reduction than LP-hydrogel (*p* < 0.05; Figure 5F). SEM observations showed relatively smooth and intact bacterial morphology in the CON-hydrogel-conditioned group, whereas exposure to LP-hydrogel-conditioned medium was associated with surface deformation and bacterial aggregation. More pronounced morphological alterations, including irregular and collapsed cell structures, were observed following exposure to LP+AVE-hydrogel-conditioned medium (Figure 5G). Collectively, these findings demonstrate that LP-containing hydrogels exhibited growth-inhibitory activity against SP and were associated with reduced attached biomass and altered bacterial morphology under the tested conditions.

### 3.6. Topical LP+AVE-Hydrogel Accelerates Closure of SP-Infected Wounds

**Figure 6:**
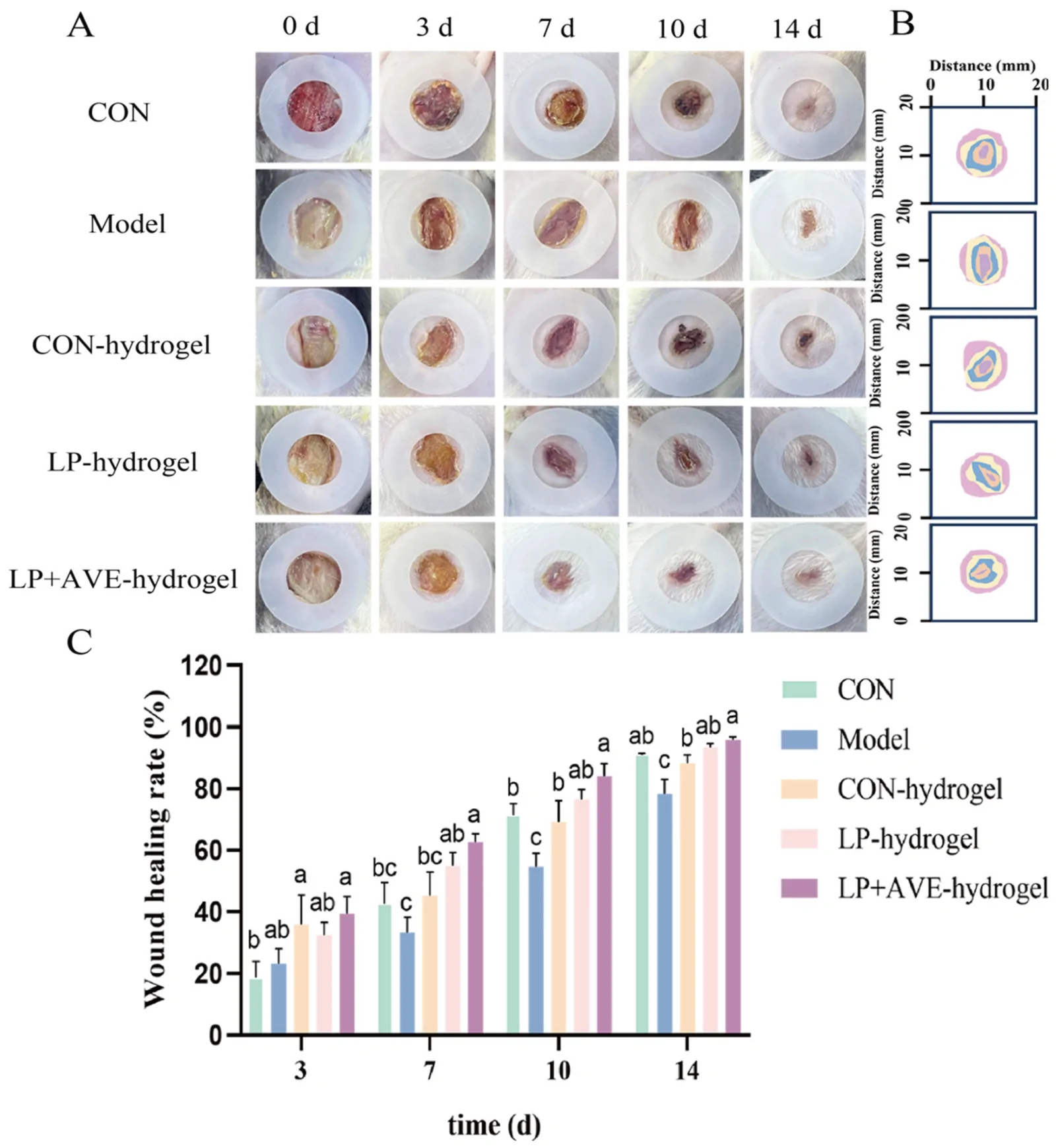
Wound-healing outcomes in SP-infected mice. (**A**) Representative photographs of wound healing in different treatment groups. (**B**) Simulated wound-area overlay maps for each treatment group. (**C**) Quantitative analysis of wound-closure rates over time. Data are presented as mean ± SEM. Each mouse had a single full-thickness dorsal wound, and the same three mice per group were repeatedly evaluated on days 0, 3, 7, 10, and 14. Different lowercase letters indicate statistically significant differences among treatment groups at the same time point (*p* < 0.05).

A full-thickness murine wound model challenged with SP was used to evaluate the therapeutic effects of the hydrogel formulations. At day 0, 48 h after SP inoculation, and immediately before treatment, infected wounds showed visible exudation and inflammatory changes consistent with the establishment of the experimental wound infection (Figure 6A). During the subsequent treatment period, the Model group showed persistent visible inflammation, scab formation, and exudation, whereas wounds treated with LP-containing hydrogels showed progressive improvement in gross appearance. Longitudinal wound-closure analysis using two-way repeated-measures ANOVA revealed a significant treatment effect (F(4, 10) = 13.139, *p* = 0.000543), as well as significant time (Greenhouse–Geisser-corrected F(2.34, 23.43) = 1091.215, *p* < 0.0001) and treatment × time interaction effects (Greenhouse–Geisser-corrected F(9.37, 23.43) = 6.860, *p* < 0.0001). These results indicate that wound-healing trajectories differed among treatment groups. By day 14, the LP+AVE-hydrogel group achieved the highest mean wound-closure rate (95.75%), significantly exceeding that of the infected Model group (78.34%; Tukey-adjusted *p* < 0.001). However, no statistically significant difference was observed between LP+AVE-hydrogel and LP-hydrogel (93.46%; *p* = 0.791). Overall, the findings indicate that LP+AVE-hydrogel treatment improved macroscopic wound closure in the SP-infected mouse model, although the additional benefit over LP-hydrogel alone was not statistically significant at day 14.

### 3.7. Qualitative Histological Features of Epidermal Coverage and Collagen Organization During Wound Repair

**Figure 7:**
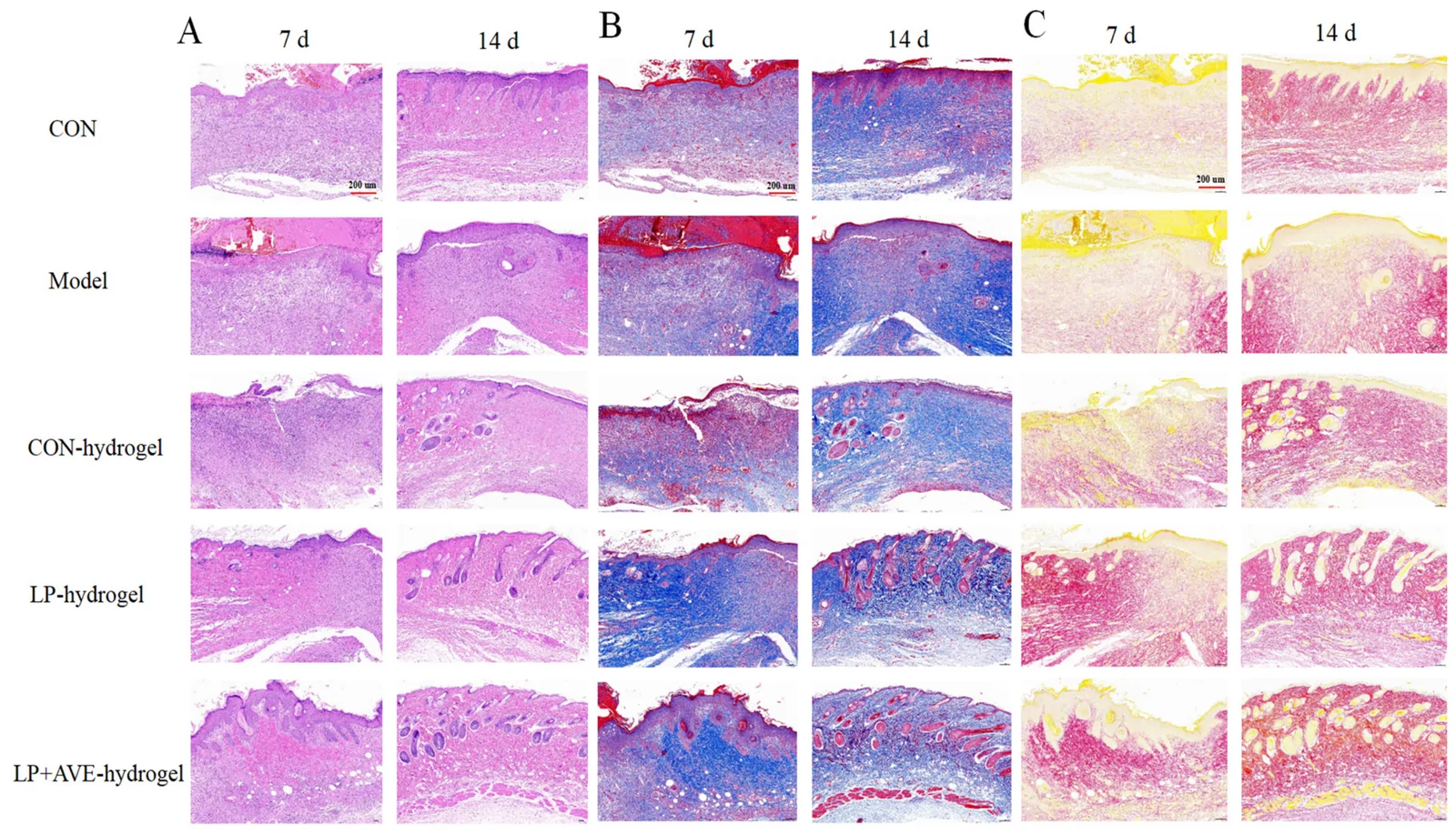
Qualitative histological evaluation of epidermal coverage and collagen-associated tissue organization during wound healing. Representative wound-tissue sections from the CON, Model, CON-hydrogel, LP-hydrogel, and LP+AVE-hydrogel groups collected on days 7 and 14. (**A**) Hematoxylin and eosin (H&E) staining showing epidermal morphology, wound-bed cellularity, and tissue organization. (**B**) Masson’s trichrome staining illustrating collagen-containing connective-tissue distribution and architecture. (**C**) Sirius Red staining showing collagen-associated extracellular-matrix distribution within the healing wound tissues.

Histological examination using hematoxylin and eosin (H&E), Masson’s trichrome, and Sirius Red staining was performed to qualitatively characterize epidermal coverage, wound-bed organization, and collagen-associated tissue architecture on days 7 and 14 (Figure 7). H&E staining revealed differences in the morphological appearance of the wound tissues among treatment groups (Figure 7A). On day 7, representative sections from the infected Model group displayed an irregular wound surface with focal crust-like material and densely cellular tissue within the wound bed, consistent with an ongoing inflammatory and reparative response. The CON-hydrogel group also exhibited an uneven epidermal surface and heterogeneous tissue organization. In contrast, sections from the LP-hydrogel and LP+AVE-hydrogel groups showed relatively continuous epidermal coverage over the examined regions and a more recognizable organization of the underlying tissue. By day 14, an epidermal covering was visible in representative sections from all groups, indicating progression of surface repair over time. Nevertheless, differences in the underlying tissue architecture remained apparent. Compared with the relatively dense and less differentiated appearance of the wound tissue in the Model group, LP-containing hydrogel-treated sections displayed more recognizable epidermal and dermal organization, with multiple adnexal-like structures visible within the examined tissue fields, particularly in the LP+AVE-hydrogel group. These observations were consistent with differences in the morphological progression of wound repair, although the extent of re-epithelialization was not quantitatively determined.

Masson’s trichrome staining was used to visualize collagen-containing connective tissue and its spatial distribution within the wound bed (Figure 7B). On day 7, blue-stained collagen-associated regions were visible in all groups but varied in distribution and continuity. Representative sections from the Model group exhibited a heterogeneous distribution of collagen-associated staining interspersed with cellular wound tissue. In LP-hydrogel- and LP+AVE-hydrogel-treated sections, blue-stained connective tissue appeared more broadly distributed in parts of the wound bed, with areas showing a relatively continuous extracellular-matrix appearance. By day 14, collagen-associated staining was prominent across the experimental groups, reflecting the presence of connective tissue within the healing wounds. The LP-containing hydrogel groups displayed recognizable collagen-rich regions interspersed with dermal cellular and adnexal structures, suggesting changes in the spatial organization of the repairing tissue. However, the staining patterns alone did not establish differences in total collagen content or collagen deposition rates. Sirius Red staining provided additional qualitative visualization of collagen-associated extracellular matrix (Figure 7C). On day 7, representative Model-group sections exhibited comparatively limited and uneven red-stained regions, whereas LP-containing hydrogel-treated sections showed more conspicuous collagen-associated staining in portions of the underlying connective tissue. On day 14, red-stained connective tissue was evident across all groups, with differences in its distribution and apparent structural arrangement. In the LP+AVE-hydrogel group, collagen-associated staining was visible throughout portions of the dermal tissue and around recognizable adnexal-like profiles, consistent with an altered organization of the extracellular matrix during wound repair. Collectively, these qualitative histological observations suggested that LP-containing hydrogel treatment was associated with differences in epidermal coverage, wound-tissue organization, and collagen-associated matrix architecture during the healing period. These morphological features were broadly consistent with the independently measured improvements in macroscopic wound closure.

### 3.8. LP+AVE-Hydrogel Attenuates Local TNF-α and Enhances IL-10 Immunoreactivity

**Figure 8:**
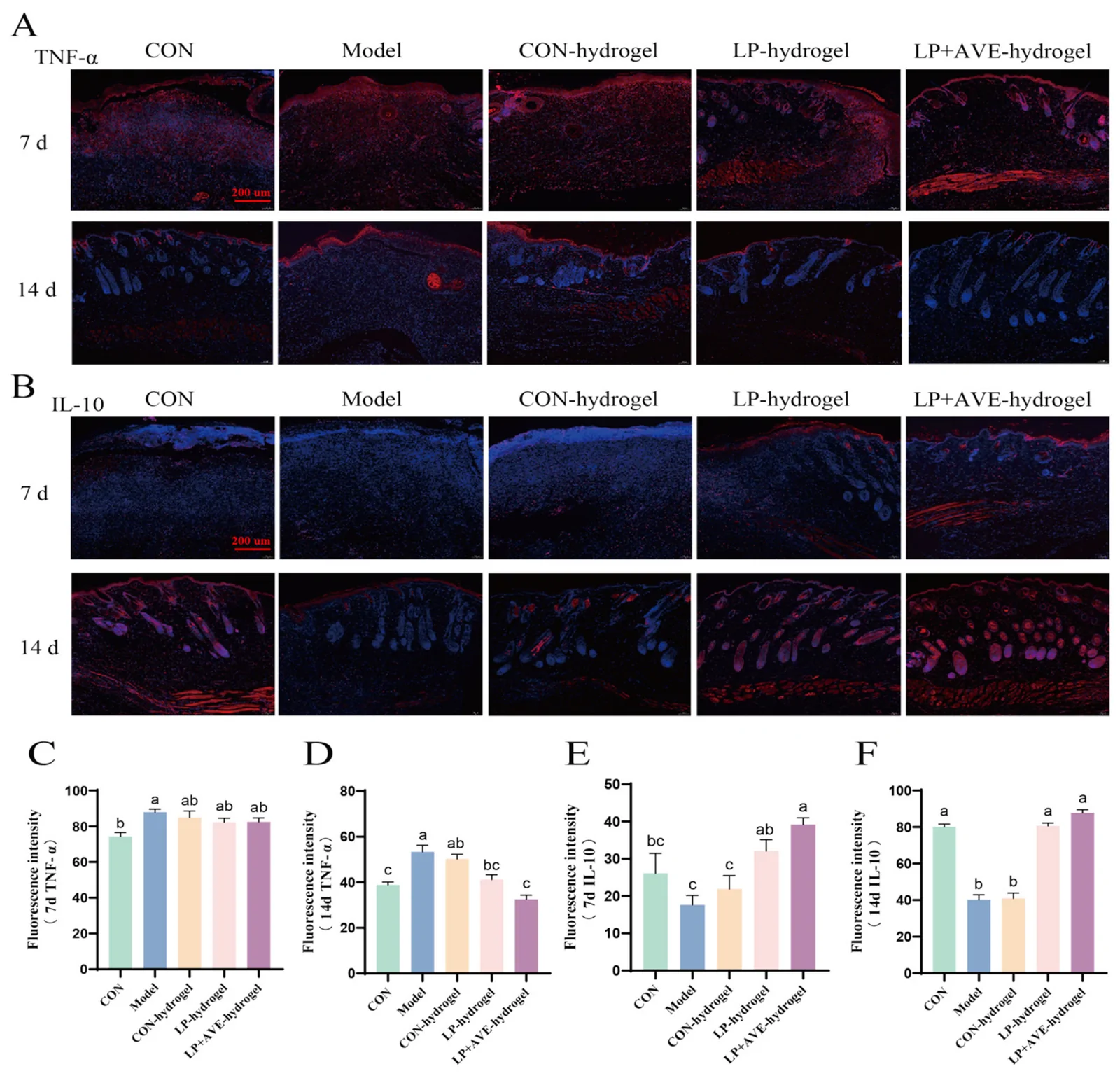
Immunofluorescence images of inflammation-related markers in skin tissue. (**A**) TNF-α immunofluorescence staining (Red) of the wound area on days 7 and 14. (**B**) IL-10 immunofluorescence staining (Red) of the wound area on days 7 and 14. (**C**,**D**) Quantification of TNF-α fluorescence intensity on days 7 and 14, respectively. (**E**,**F**) Quantification of IL-10 fluorescence intensity on days 7 and 14, respectively. The data are presented as mean ± sem, and different letters show significant differences (*p* < 0.05).

Immunofluorescence staining was used to characterize local inflammatory responses in wound tissues on days 7 and 14. On day 7, TNF-α immunoreactivity was evident in the Model group (Figure 8A). Although the LP-hydrogel and LP+AVE-hydrogel groups exhibited numerically lower mean TNF-α fluorescence intensities, neither treatment group differed significantly from the Model group (*p* > 0.05; Figure 8C). In contrast, IL-10 immunoreactivity was relatively weak in the Model and CON-hydrogel groups but increased following LP-containing hydrogel treatment, with the strongest signal observed in the LP+AVE-hydrogel group (Figure 8B,E). By day 14, TNF-α fluorescence intensity was significantly lower in both the LP-hydrogel and LP+AVE-hydrogel groups than in the Model group (*p* < 0.05; Figure 8D). IL-10 immunoreactivity was also higher in the LP-containing hydrogel groups than in the Model group at this time point (*p* < 0.05; Figure 8F). These findings indicate that LP+AVE-hydrogel was associated with attenuation of local TNF-α immunoreactivity and enhancement of IL-10 during infected-wound repair.

### 3.9. LP+AVE-Hydrogel Shifts CD80/CD206 Immunoreactivity Toward a Reparative-Associated Profile

**Figure 9:**
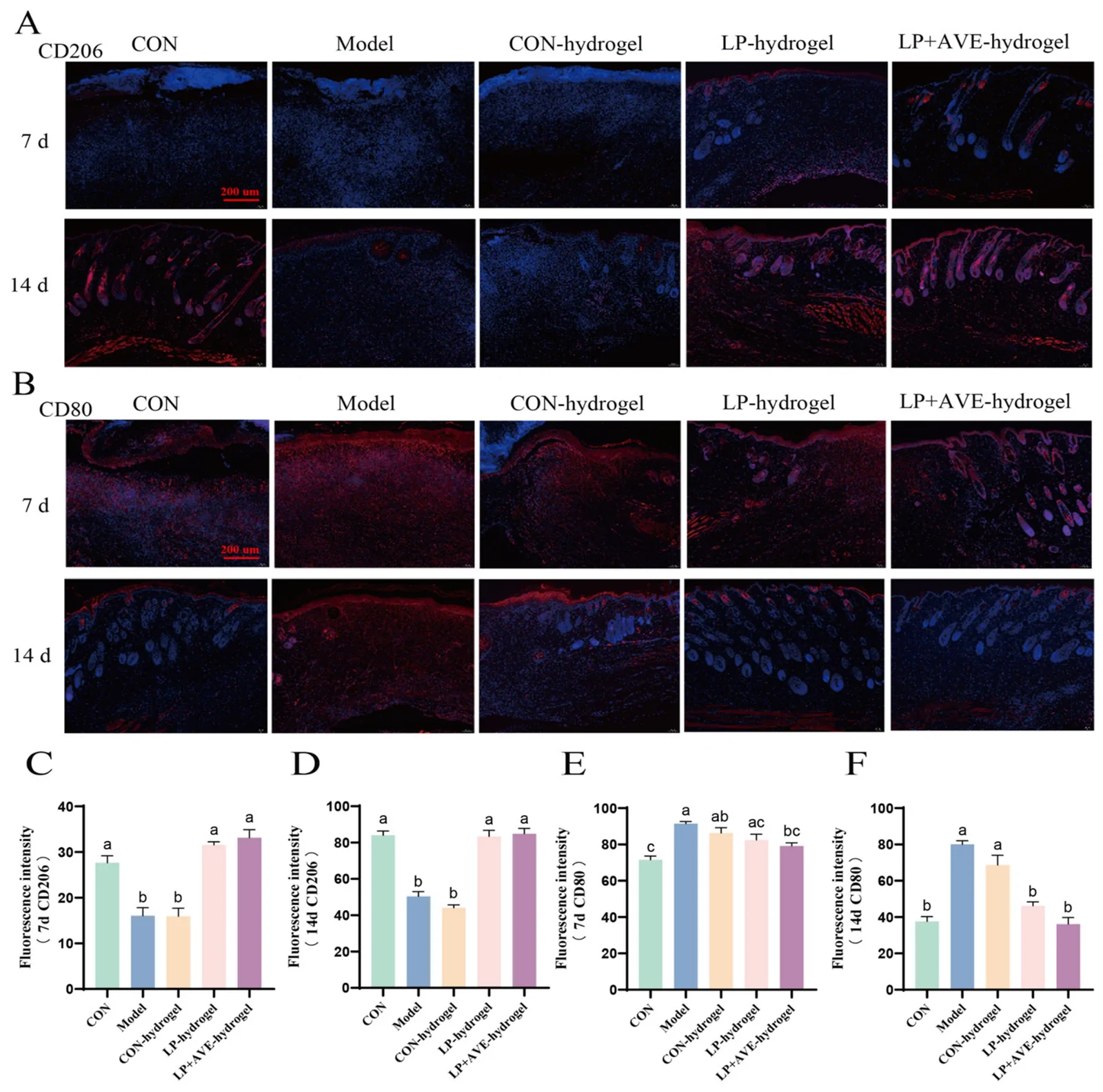
Immunofluorescence images of macrophage markers in skin tissue. (**A**) CD206 immunofluorescence staining (Red) in the wound area on days 7 and 14; (**B**) CD80 immunofluorescence staining (Red) in the wound area on days 7 and 14. (**C**,**D**) Quantification of CD206 fluorescence intensity on days 7 and 14, respectively. (**E**,**F**) Quantification of CD80 fluorescence intensity on days 7 and 14, respectively. The data are presented as mean ± sem, and different letters show significant differences (*p* < 0.05).

CD206 and CD80 immunofluorescence were assessed to characterize macrophage-associated marker patterns during wound healing. On day 7, CD206 immunoreactivity was relatively weak in the Model and CON-hydrogel groups but was increased in LP-hydrogel and LP+AVE-hydrogel-treated wounds (Figure 9A). Quantitative analysis showed significantly greater CD206 fluorescence intensity in the probiotic-treated groups, with the highest signal observed in the LP+AVE-hydrogel group (*p* < 0.05; Figure 9C).

Conversely, CD80 immunoreactivity was prominent in the Model group and remained relatively high in the CON-hydrogel group. Treatment with LP-hydrogel reduced the CD80 signal, whereas LP+AVE-hydrogel produced a further reduction (Figure 9B,E). By day 14, CD206 immunoreactivity remained higher and CD80 immunoreactivity lower in the LP-containing hydrogel groups than in the Model and CON-hydrogel groups (Figure 9A–F; *p* < 0.05). Thus, LP+AVE-hydrogel treatment was associated with a shift in CD80/CD206 marker patterns toward a profile consistent with tissue repair.

### 3.10. LP+AVE-Hydrogel Modulates Systemic Inflammatory and Repair-Associated Mediators

**Figure 10:**
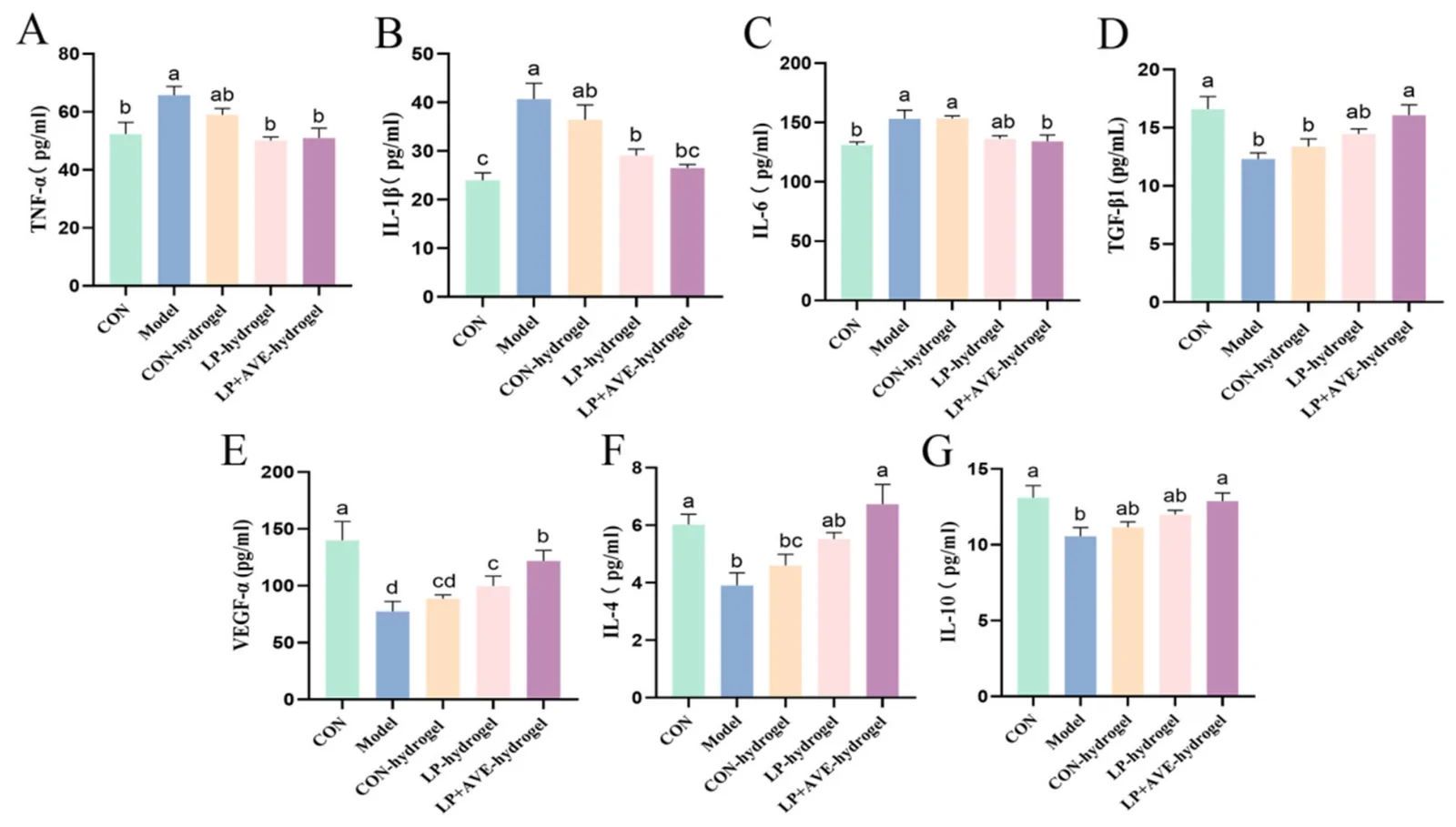
Effects of different hydrogels on serum inflammatory parameters in mice at day 14. (**A**) TNF-α, (**B**) IL-1β, (**C**) IL-6, (**D**) TGF-β1, (**E**) VEGF-α, (**F**) IL-4, (**G**) IL-10. The data are presented as mean ± SEM, and different letters show significant differences (*p* < 0.05).

Serum inflammatory and tissue-repair-related mediators were measured on day 14 to assess systemic responses to treatment. Compared with the Model group, LP+AVE-hydrogel significantly reduced serum TNF-α, IL-1β, and IL-6 concentrations (*p* < 0.05; Figure 10A–C). In parallel, LP+AVE-hydrogel treatment was associated with significantly increased serum concentrations of IL-4, IL-10, TGF-β1, and VEGF compared with the Model group (Figure 10D–G; *p* < 0.05). The increase in serum VEGF represents a change in a vascular-related signaling factor and should not be interpreted as direct evidence of angiogenesis For several measured mediators, no significant difference was detected between LP+AVE-hydrogel and the non-infected CON group, indicating that the corresponding values approached those observed in non-infected animals. Collectively, these findings show that LP+AVE-hydrogel reduced systemic pro-inflammatory mediators while increasing anti-inflammatory and repair-associated factors during SP-infected wound healing.

### 3.11. LP+AVE-Hydrogel Is Associated with Remodeling of the Infected-Wound Microbiota

**Figure 11:**
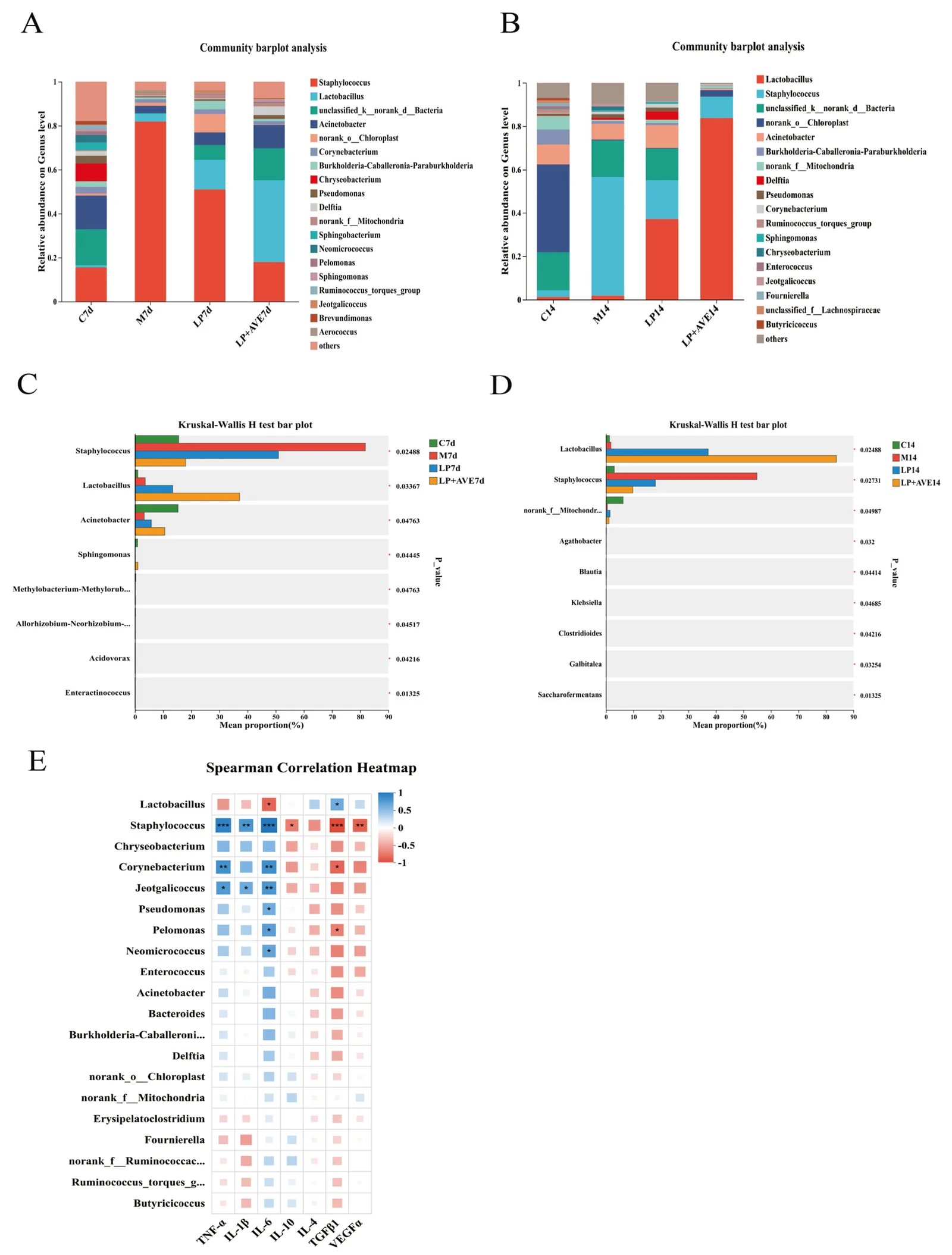
Wound microbiota analysis during wound healing. (**A**,**C**) Genus-level microbial community composition on day 7. (**B**,**D**) Genus-level microbial community composition on day 14. (**E**) Spearman correlation heatmap showing associations between wound microbiota and serum parameters. All samples passed sample-level quality control and were retained for downstream analysis. Statistical significance is indicated as * *p* < 0.05, ** *p* < 0.01, and *** *p* < 0.001.

Wound-tissue microbiota were analyzed by 16S rRNA gene sequencing on days 7 and 14 to evaluate treatment-associated changes in the local microbial community. Genus-level community profiles differed among the CON, Model, LP-hydrogel, and LP+AVE-hydrogel groups (Figure 11A,B). Compared with the Model group, LP-hydrogel and LP+AVE-hydrogel were associated with lower relative abundance of Staphylococcus and higher relative abundance of Lactobacillus, with the largest shift observed in the LP+AVE-hydrogel group (Figure 11C,D). These findings indicate that probiotic hydrogel treatment was associated with changes in the microbial composition of the infected wound. Additional exploratory analyses of wound microbial diversity are presented in Supplementary Figure S1. The ACE, Chao1, Shannon, and Simpson indices were used to characterize alpha-diversity patterns, while genus-level PCoA illustrated differences in microbial community distribution among experimental groups on days 7 and 14.

Exploratory Spearman correlation analysis was performed using 12 individually matched wound microbiota and serum samples collected on day 14, pooled across the four experimental groups. Staphylococcus relative abundance showed positive correlations with several pro-inflammatory mediators, including TNF-α, IL-1β, and IL-6, whereas Lactobacillus relative abundance generally exhibited opposite correlation patterns with inflammatory and repair-associated parameters (Figure 11E). However, because observations from different treatment groups were combined, these associations may partly reflect treatment-related differences in both microbial composition and host responses. Therefore, the observed correlations should be interpreted as exploratory associations rather than evidence of direct or treatment-independent microbial effects.

## 4. Discussion

*Staphylococcus pseudintermedius* is an important opportunistic pathogen in companion-animal medicine, particularly in canine skin and soft-tissue infections, and its clinical management has become increasingly complicated by antimicrobial resistance and biofilm formation. Methicillin-resistant S. pseudintermedius frequently exhibits resistance to multiple antimicrobial classes, while biofilm-forming isolates are commonly recovered from canine pyoderma and other skin lesions [2,3,5,6,7,8]. The increasing recognition of SP colonization and infection in humans, together with evidence of probable transmission between dogs and their owners, further extends its relevance to a One Health context [10,11,12]. Against this background, the present study developed an LP-centered topical formulation incorporating feline-derived *Lactiplantibacillus plantarum* ZYpet-014, a commercial Aloe vera-derived preparation (AVE), and a carbomer-based delivery matrix. The LP-containing formulations exhibited growth-inhibitory activity against SP in vitro and were associated with improved wound-healing outcomes in an experimentally infected murine model. These effects were accompanied by changes in inflammatory mediators, repair-associated tissue features, CD80/CD206 immunoreactivity, and wound microbial composition. Notably, 16S rRNA gene sequencing revealed treatment-associated changes in the relative abundance of Staphylococcus and other bacterial genera. However, genus-level relative abundance does not provide a direct measure of absolute SP burden, and species-specific pathogen quantification was not performed in wound tissues. Therefore, the present findings cannot establish SP clearance in vivo or demonstrate that the observed microbial changes were causally responsible for wound repair. Rather, they provide preliminary evidence linking the topical formulation with altered host responses and wound microbial community composition.

A distinctive feature of the formulation was the incorporation of AVE as a potential functional adjunct to LP, although the independent therapeutic contribution of AVE was not evaluated in the present study. In vitro experiments showed that AVE supplementation enhanced LP growth within a defined concentration range and was associated with increased endpoint concentrations of several organic acids in culture supernatants. However, these observations do not establish enhanced metabolic activity per bacterial cell or demonstrate that AVE exerts its effects exclusively through supporting LP. Importantly, an AVE-only hydrogel group was not included in the present study; therefore, the independent antibacterial, anti-inflammatory, or wound-healing contributions of AVE cannot be excluded, and a synergistic interaction between AVE and LP cannot be established. Previous studies have reported prebiotic-related and other biological activities of purified acemannan and Aloe-derived polysaccharides [25,31,32]. Similar principles have been reported in living probiotic hydrogel systems in which polysaccharide-containing matrices supported probiotic growth and metabolic output [21,22]. Nevertheless, the AVE used in this study was a commercially supplied Aloe vera-derived preparation with a supplier-declared purity of at least 60%, and its detailed polysaccharide composition, molecular-weight distribution, and nonpolysaccharide constituents were not independently characterized. Consequently, the biological activities reported for purified acemannan cannot be directly attributed to the AVE preparation used here. The present findings support further investigation of AVE as a potential component of LP-centered topical formulations, while additional component-controlled studies are required to determine its independent and LP-mediated contributions. The carbomer matrix served as a hydrated vehicle for the topical incorporation of LP. Initial viable-cell counting confirmed the presence of culturable LP in freshly prepared formulations, while SYTO 9/PI staining after 48 h of storage at 4 °C revealed relatively favorable membrane-integrity-associated fluorescence characteristics in the LP-containing hydrogels. The tube-inversion test, SEM observations, and swelling measurements further provided preliminary evidence of gel formation and relevant physical characteristics for experimental application. Previous studies have demonstrated the potential of hydrogel matrices to support probiotic delivery and bacterial survival [20,21,34]. Nevertheless, the fluorescence-based measurements were not validated against post-storage culturable counts, and neither LP persistence nor metabolic activity following topical application was directly assessed. Furthermore, these preliminary evaluations do not establish long-term formulation stability, quantitative rheological performance, or retention under physiological wound conditions. Accordingly, the present findings support the preliminary feasibility of carbomer as an experimental topical carrier rather than demonstrating sustained probiotic delivery or the suitability of a fully characterized, clinically optimized wound formulation.

Continued metabolic activity of LP was accompanied by organic-acid production and acidification of the surrounding medium. AVE supplementation increased LP-associated organic-acid production, particularly lactic and acetic acids, while LP-containing hydrogels progressively reduced the pH of the surrounding PBS. Given the importance of environmental pH in bacterial growth and wound repair [42,43,44], acidification may contribute to the antibacterial activity of the formulations. However, the organic-acid measurements, hydrogel-associated pH measurements, and antibacterial assays were conducted under different experimental conditions, and no pH-matched or neutralized conditioned-medium controls were included. Therefore, the contribution of acidification to SP inhibition cannot be determined. The low hemolysis rates of the hydrogel formulations indicated favorable hemocompatibility under the tested conditions. The in vitro assays demonstrated growth-inhibitory activity of LP-containing hydrogels against SP, with LP+AVE-hydrogel generally exhibiting greater effects in the agar-diffusion and endpoint viable-counting assays. Crystal-violet staining also revealed a greater reduction in attached biomass following treatment with LP+AVE-hydrogel. However, this assay evaluated biomass accumulation during co-incubation with the formulations rather than the removal of pre-established biofilms. Moreover, the staining was not species-specific, and potential contributions from LP-associated biomass and hydrogel components could not be independently resolved. Therefore, these findings should not be interpreted as evidence of mature SP biofilm eradication or control of persistent infection. Previous studies have reported antimicrobial and biofilm-related effects of L. plantarum and its derivatives, potentially involving organic acids, bacteriocin-like compounds, and microbial competition [13,14,15]. LP-derived preparations and probiotic-containing dressings have also shown activity against staphylococcal pathogens in experimental wound models [14,18,19]. Nevertheless, these potential mechanisms were not individually investigated in the present study. Thus, although the LP-containing formulations exhibited antibacterial and antibiofilm activity, the relative contributions of acidification and other LP-derived antimicrobial factors remain unresolved.

The greater antibacterial and antibiofilm activity observed for LP+AVE-hydrogel compared with LP-hydrogel indicates that AVE incorporation improved the overall performance of the composite formulation under the tested conditions. The ability of AVE to promote LP proliferation and organic-acid production in vitro provides a plausible explanation for this improvement. However, Aloe-derived polysaccharides and Aloe-containing materials have also been reported to possess antibacterial properties [27,28], suggesting that AVE may independently contribute to the activity of the combined formulation. Therefore, the enhanced antibacterial and antibiofilm effects observed with LP+AVE-hydrogel may reflect AVE-mediated enhancement of LP activity, independent AVE effects, or contributions from both components. Because an AVE-only hydrogel group was not included, the relative contributions of these potential mechanisms cannot be determined. Nevertheless, these findings support the improved antibacterial performance of the combined formulation without establishing a specific interaction mechanism or demonstrating synergistic activity between LP and AVE. In vivo, LP+AVE-hydrogel treatment was associated with improvements in wound repair, although direct suppression of SP within infected wound tissues was not quantitatively established. In the SP-infected murine wound model, treatment with LP-containing hydrogels was associated with improved macroscopic wound closure and changes in wound-tissue morphology. Representative H&E-stained sections suggested relatively continuous epidermal coverage and more recognizable underlying tissue organization in LP-containing hydrogel-treated wounds, while Masson’s trichrome and Sirius Red staining revealed qualitative differences in collagen-associated staining patterns. These observations were consistent with the wound-closure measurements but were primarily qualitative and were not supported by quantitative histomorphometric assessment of collagen deposition or tissue remodeling. Cutaneous wound repair involves coordinated inflammatory, proliferative, and remodeling processes [35], and previous studies have reported improvements in wound healing following LP-based interventions [13,17,18,45]. Nevertheless, the present study assessed wound repair and inflammatory responses during the same treatment course. It therefore cannot distinguish direct host immunomodulatory effects from changes secondary to improvements in the overall course of wound healing or infection. Furthermore, because absolute SP burden was not quantified in wound tissues, the contribution of in vivo pathogen reduction to the observed repair responses remains unresolved.

Aloe-derived materials have also been investigated for their potential effects on cutaneous repair. Previous studies reported that purified acemannan promoted cell proliferation and wound healing through AKT/mTOR-associated signaling [24], whereas a characterized Aloe polysaccharide preparation influenced keratinocyte proliferation, migration, and differentiation through EGFR/PKC-dependent pathways [26]. Other Aloe-containing preparations have been associated with collagen-related and wound-repair responses [28,29,30]. However, these findings cannot be directly extrapolated to the present formulation. The AVE used in this study was a commercial Aloe vera-derived preparation with a supplier-declared purity of at least 60%, and its detailed chemical composition was not independently characterized or demonstrated to be equivalent to the materials investigated in those studies. Moreover, neither AKT/mTOR nor EGFR/PKC signaling was examined experimentally. Accordingly, these pathways are discussed as potential mechanisms reported in the literature rather than as demonstrated mediators of the effects observed with LP+AVE-hydrogel. The histological findings provided complementary evidence of tissue repair following LP-containing hydrogel treatment. H&E staining revealed a more continuous epidermal appearance and better-organized wound-tissue architecture in representative LP+AVE-treated sections, although epidermal coverage was also evident in the untreated infected group by day 14. Masson’s trichrome and Sirius Red staining further suggested differences in the distribution and organization of collagen-containing extracellular matrix, consistent with ongoing tissue remodeling. These qualitative observations were broadly consistent with the improved wound-closure outcomes and changes in repair-associated mediators. However, quantitative histomorphometric and collagen-specific analyses were not performed; therefore, the findings cannot establish the extent of re-epithelialization, collagen deposition, or collagen maturation. Inflammatory regulation appears to be another important component of this response. Persistent infection can prolong inflammatory signaling and interfere with epithelial repair and extracellular-matrix reconstruction [35,46]. Changes in inflammatory and repair-associated markers accompanied the wound-healing response. LP+AVE-hydrogel treatment was associated with lower TNF-α immunoreactivity and higher IL-10 immunoreactivity in wound tissue, together with reduced circulating TNF-α, IL-1β, and IL-6 and increased IL-4, IL-10, TGF-β1, and VEGF. These findings indicate treatment-associated differences in local inflammatory-marker expression and systemic mediator concentrations. However, they do not establish whether the changes resulted from direct immune modulation or occurred secondarily during wound repair. In particular, elevated circulating VEGF and TGF-β1 cannot independently demonstrate local angiogenesis, quantitative collagen remodeling, or functional tissue recovery, as vessel-specific, perfusion-based, and functional assessments were not performed. Previous studies of LP-based wound interventions have described changes in inflammatory and repair-associated mediators [13,14,21], providing a relevant biological context. Overall, the present findings support an association between LP-containing hydrogel treatment, improved wound closure, and altered inflammatory and repair-associated markers, while the underlying causal mechanisms require further investigation.

Changes in CD80 and CD206 immunoreactivity provided additional information on the local tissue environment during wound repair. LP+AVE-hydrogel treatment was associated with lower CD80-positive staining and higher CD206-positive staining in wound sections, consistent with changes in the distribution of inflammation- and repair-associated markers. Macrophages participate in inflammatory regulation, extracellular-matrix remodeling, and tissue repair [35,46], and previous studies have reported changes in macrophage-associated responses following LP- or Aloe-containing interventions [17,29,30,47]. However, CD80 and CD206 are not exclusive markers of macrophages, and their expression alone cannot establish macrophage identity, functional polarization, or direct immunomodulation. Moreover, differences in positive-staining area may reflect changes in inflammatory-cell infiltration, tissue cellularity, or the relative proportions of different cell populations during healing, rather than altered marker expression within a defined macrophage population. Because macrophage-specific co-localization and detailed cellular phenotyping were not performed, the observed CD80/CD206 patterns are best interpreted as treatment-associated changes in wound-tissue immunoreactivity rather than definitive evidence of macrophage reprogramming.

The wound microbiota analysis similarly revealed treatment-associated differences in bacterial community composition. LP-containing hydrogel treatment was associated with a higher relative abundance of Lactobacillus and a lower relative abundance of Staphylococcus than the infected Model group. Previous studies have highlighted the potential of probiotic-containing hydrogels to influence wound microbial communities [21,22], while interactions between wound-associated microorganisms and host responses are recognized as important aspects of tissue repair [36,37,38,39,40]. Nevertheless, the increased relative abundance of Lactobacillus could reflect the administered LP strain, indigenous bacteria belonging to this genus, or residual bacterial DNA, and genus-level 16S rRNA gene sequencing could not distinguish these possibilities or establish viable LP persistence. Likewise, the lower relative abundance of Staphylococcus may partly reflect compositional changes in the microbial community and cannot establish a reduction in absolute SP burden. Consequently, the observed taxonomic differences do not demonstrate restoration of microbial balance or pathogen clearance. Exploratory Spearman correlations between wound bacterial genera and serum inflammatory or repair-associated mediators were calculated using 12 matched observations from four treatment groups on day 14. Because these observations were pooled across groups, the associations may have been influenced by treatment allocation, differences in healing status, and the compositional nature of relative-abundance data. Therefore, these correlations do not independently establish that microbial changes regulate host inflammatory responses. Collectively, the findings describe concurrent treatment-associated differences in wound-tissue marker patterns and microbial composition, providing a basis for further investigation of their potential relationships during wound repair.

From a veterinary perspective, this integrated activity is particularly relevant because current management of SP infection is increasingly constrained by antimicrobial resistance. MRSP frequently exhibits resistance to multiple drug classes, narrowing treatment options in small-animal practice [2,3,4,5]. Probiotic, prebiotic, synbiotic, and postbiotic wound formulations have consequently attracted interest as alternative local strategies, although most current evidence remains preclinical [15,16,34]. The zoonotic potential of SP and documented evidence of dog–human transmission further strengthen the One Health relevance of developing additional infection-control approaches [10,11,12]. The novelty of the present study lies in combining a feline-derived LP strain, a commercial Aloe vera-derived preparation, and a carbomer-based hydrogel in a topical formulation evaluated against SP. The hydrogel matrix enabled the incorporation of culturable LP, while fluorescence staining after short-term storage provided preliminary information on bacterial membrane-integrity-associated characteristics. However, sustained probiotic viability and metabolic activity following topical application were not established. In vitro, the LP-containing formulations exhibited growth-inhibitory activity against SP and were associated with reduced crystal-violet-stained attached biomass. In the murine wound model, LP+AVE-hydrogel treatment was associated with improved wound closure, changes in inflammatory and repair-associated markers, qualitative differences in wound-tissue organization, and alterations in the relative abundance of selected bacterial genera. Although these findings describe multiple treatment-associated responses, they do not establish causal or coordinated interactions among pathogen inhibition, host inflammatory responses, and microbial community changes. Overall, the present study provides preliminary proof-of-concept evidence supporting the potential of an LP-containing topical formulation for SP-associated wound infection, while the underlying mechanisms and sustained probiotic activity at the wound site require further investigation.

Several limitations should be acknowledged. First, the present study was primarily designed to evaluate the therapeutic potential of an LP-centered topical formulation, with AVE incorporated as a potential functional adjunct. However, an AVE-only hydrogel group was not included. Given the reported antibacterial, anti-inflammatory, and wound-healing activities of Aloe vera polysaccharides [24,25,26,27,28,29,30], the independent contribution of AVE to the observed therapeutic effects cannot be excluded. Although AVE enhanced LP growth and organic-acid production, these findings do not establish that the improved performance of LP+AVE-hydrogel resulted exclusively from enhanced probiotic activity or demonstrate a synergistic interaction between the two components. Furthermore, because pH-matched and neutralized conditioned-medium controls were not included, the relative contributions of acidification and other LP-derived antimicrobial factors could not be distinguished. Future studies incorporating appropriate component controls and pH-controlled experiments will be necessary to clarify these mechanisms.

Second, the absence of direct quantification of SP in infected wound tissues limits the interpretation of the in vivo antibacterial effects. Although the formulation inhibited SP growth and biofilm formation in vitro, the genus-level relative-abundance data obtained from 16S rRNA gene sequencing cannot establish changes in absolute SP burden or pathogen clearance. Similarly, increased Lactobacillus relative abundance does not demonstrate persistence or colonization by the administered ZYpet-014 strain. The relatively small number of biological replicates used for microbiota analysis also limits statistical power and generalizability. Moreover, exploratory correlations between microbial taxa and inflammatory parameters may have been influenced by treatment-related differences because observations from different treatment groups were pooled. Future investigations incorporating species-specific pathogen quantification, strain-specific probiotic tracking, and larger biological cohorts would strengthen these findings.

Finally, further characterization of both the probiotic strain and the hydrogel formulation is required before clinical translation. Preliminary phenotypic antimicrobial susceptibility screening and targeted PCR analyses of selected virulence-associated and antibiotic-resistance genes were conducted for ZYpet-014, and the corresponding results are provided in the Supplementary Materials. However, these investigations were limited to disk-diffusion screening and five selected genetic targets. The resulting susceptibility categories were not established using validated, strain-appropriate interpretive criteria, and the absence of detectable amplification for the selected genes does not exclude other resistance or virulence-associated determinants. Accordingly, standardized MIC testing and whole-genome-based characterization, including assessment of acquired resistance genes and mobile genetic elements, remain necessary before clinical translation. In addition, the antibacterial activity was evaluated against a reference SP strain rather than a panel of clinically relevant isolates with defined resistance profiles. From a formulation perspective, the present study provided preliminary assessments of gel formation, swelling behavior, short-term probiotic viability, and hemocompatibility. However, longer-term storage stability and viable-cell retention, quantitative rheological properties, topical adhesion and retention, and cytocompatibility with keratinocytes and dermal fibroblasts were not systematically evaluated. These properties, together with long-term topical safety and therapeutic efficacy in naturally occurring canine or feline infections, should be investigated in future studies.

Despite these limitations, the present findings provide proof-of-concept evidence supporting the potential of a feline-derived LP+AVE-hydrogel as a non-antibiotic topical approach for SP-associated wound infection.

## 5. Conclusions

In conclusion, the LP+AVE-hydrogel inhibited SP growth and reduced crystal-violet-stained attached biomass under the tested in vitro conditions. In a murine model of SP-infected cutaneous wounds, topical LP+AVE treatment was associated with improved macroscopic wound closure, differences in local and systemic inflammatory and repair-associated markers, and qualitative changes in wound-tissue organization. Treated wounds also exhibited altered CD80/CD206 immunoreactivity and changes in the genus-level relative abundances of Staphylococcus and Lactobacillus. These findings provide preliminary evidence supporting the potential of a feline-derived LP-containing hydrogel as a non-antibiotic topical approach for SP-associated wound management. However, sustained probiotic viability and metabolic activity at the wound site, absolute reduction in SP burden in vivo, and causal relationships among microbial changes, inflammatory responses, and wound repair were not established. Further studies using antimicrobial-resistant clinical SP isolates and naturally occurring canine or feline infections are needed before clinical translation.

## Data Availability

The original contributions presented in this study are included in the article/Supplementary Materials. Further inquiries can be directed to the corresponding author.

## Supplementary Materials

The following supporting information can be downloaded at: https://www.mdpi.com/article/10.3390/vetsci13101126/s1, Figure S1: Exploratory alpha- and beta-diversity analyses of wound microbial communities; Figure S2: Phylogenetic position of *Lactiplantibacillus plantarum* ZYpet-014 inferred from 16S rRNA gene sequences; Table S1: Antibiotic susceptibility screening of *Lactiplantibacillus plantarum* ZYpet-014; Table S2: Targeted screening of virulence-associated and antibiotic-resistance genes in ZYpet-014; Methods S1: Identification and Preliminary Safety-Related Characterization of *Lactiplantibacillus plantarum* ZYpet-014.
