# Supplementary material for: Feline-Derived Lactiplantibacillus plantarum–Aloe Vera Polysaccharide Hydrogel as a Topical Control Strategy for Staphylococcus pseudintermedius-Infected Wounds
Source: Vet Sci. 2026 Oct 21;13(10):1126. doi: 10.3390/vetsci13101126 (PMC13709649; doi:10.3390/vetsci13101126)
Supplement: Supplementary file 1 [file vetsci-13-01126-s001.zip › vetsci-4612476-supplementary.pdf]

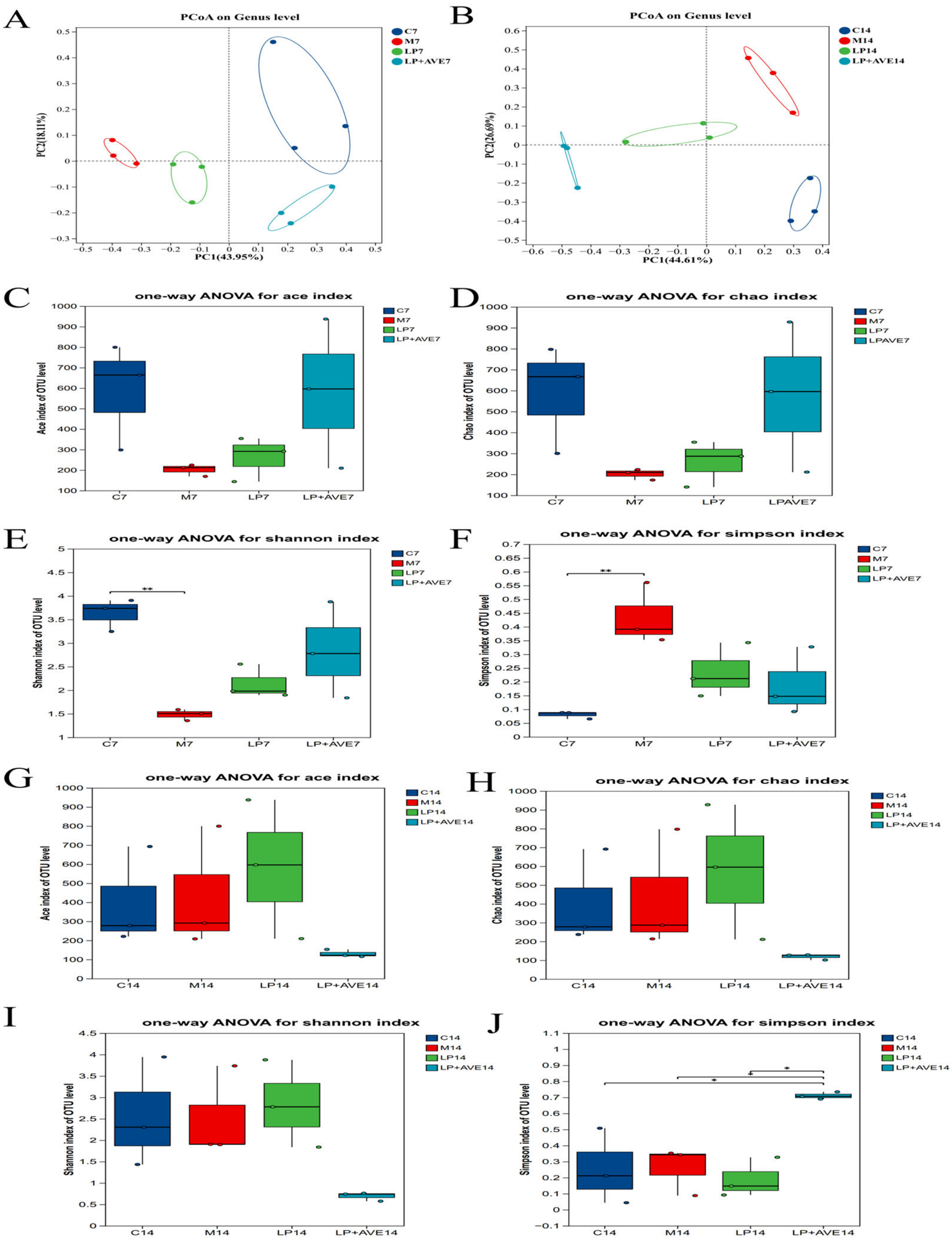

Supplementary Figure S1. Exploratory alpha- and beta-diversity analyses of wound microbial communities. (A,B) Principal coordinate analysis (PCoA) of genus-level wound microbial communities on days 7 and 14, respectively. (C–F) ACE, Chao1, Shannon, and Simpson alpha-diversity indices on day 7. (G–J) Corresponding alpha-diversity indices on day 14. Four experimental groups were included: CON, Model, LP-hydrogel, and LP+AVE-hydrogel, with three independent biological replicates per group at each sampling time point (n = 3). PCoA plots provide descriptive visualization of between-sample community variation and do not independently establish statistically significant differences in overall microbial community structure. Statistical annotations in the alpha-diversity plots represent comparisons from the original exploratory analyses. These results should be interpreted cautiously because of the limited biological sample size.

Supplementary Methods S1. Identification and Preliminary Safety-Related Characterization of Lactiplantibacillus plantarum ZYpet-014

S1.1. Molecular Identification of ZYpet-014

The feline-derived strain ZYpet-014 was characterized by 16S rRNA gene sequence analysis. The obtained 16S rRNA gene sequence was compared with reference sequences of phylogenetically related lactic acid bacterial species retrieved from the NCBI GenBank database. Multiple sequence alignment was performed using the MUSCLE algorithm implemented in MEGA11 software (version 11.0). A phylogenetic tree was constructed using the neighbor-joining method based on the Kimura two-parameter model. The reliability of the inferred phylogenetic relationships was evaluated by bootstrap analysis with 1,000 replicates. Bacillus subtilis ATCC 21332 was included as the outgroup. The resulting phylogenetic tree was used to assess the taxonomic position of ZYpet-014 relative to the type strain Lactiplantibacillus plantarum JCM 1149^{T} and other closely related species.

S1.2. Antibiotic Susceptibility Testing

The antibiotic susceptibility of Lactiplantibacillus plantarum ZYpet-014 was evaluated using a modified disk-diffusion method. Ten antimicrobial agents were examined using commercially prepared antibiotic-impregnated disks: gentamicin (GEN, 10 μg/disk), ciprofloxacin (CIP, 5 μg/disk), ceftriaxone (CTR, 30 μg/disk), erythromycin (E, 15 μg/disk), ampicillin (AMP, 10 μg/disk), tetracycline (TET, 30 μg/disk), trimethoprim–sulfamethoxazole (SXT, 25 μg/disk), chloramphenicol (C, 30 μg/disk), lincomycin (MY, 2 μg/disk), and penicillin (PEN, 10 U/disk). ZYpet-014 was cultured in de Man, Rogosa and Sharpe (MRS) broth at 37 °C for 18–24 h. The bacterial suspension was adjusted to a turbidity equivalent to the 0.5 McFarland standard (approximately 1.5×10^{8} CFU/mL). The suspension was subsequently diluted tenfold in sterile phosphate-buffered saline (PBS, pH 7.4), and 100 μL of the diluted suspension was evenly spread onto MRS agar plates. Antibiotic disks were aseptically placed on the inoculated agar surface, and the plates were incubated at 37 °C for 48 h. Following incubation, the diameters of the inhibition zones, including the antibiotic disks, were measured in millimeters. Each assay was performed in triplicate, and the results were expressed as the mean ± standard deviation. Because standardized disk-diffusion breakpoints have not been established for ZYpet-014 under these experimental conditions, inhibition-zone measurements were considered descriptive indicators of antimicrobial susceptibility rather than definitive clinical susceptibility classifications. The results are presented in Supplementary Table S1.

S1.3. PCR Screening of Selected Virulence-Associated and Antibiotic-Resistance Genes

The presence of selected virulence-associated and antibiotic-resistance genes in Lactiplantibacillus plantarum ZYpet-014 was investigated using conventional polymerase chain reaction (PCR). Five genetic determinants were examined, including three virulence-associated genes, ace (collagen-binding protein), gelE (gelatinase), and cylA (cytolysin-associated gene), and two antibiotic-resistance genes, vanA (vancomycin resistance) and tetM (tetracycline resistance). Genomic DNA was extracted from an overnight culture of ZYpet-014 using a bacterial genomic DNA extraction kit according to the manufacturer's instructions. The extracted DNA was used as the template for PCR amplification with gene-specific primers. The primer sequences and expected amplification product sizes are provided in Supplementary Table S2. PCR amplification was performed in a total reaction volume of 25 μL containing 12.5 μL of 2× PCR Master Mix, 1.0 μL of each primer (10 μM), 1.0 μL of genomic DNA template (approximately 20–50 ng), and 9.5

μL of nuclease-free water. Amplification was performed using the following thermal cycling conditions: initial denaturation at 95 °C for 5 min, followed by 35 cycles of denaturation at 95 °C for 30 s, annealing at 55 °C for 30 s, and extension at 72 °C for 60 s, with a final extension at 72 °C for 5 min. Target-verified positive-control DNA was used to assess the amplification performance of each gene-specific PCR assay. Enterococcus faecalis ATCC 29212 was used as a reference strain for the virulence-associated gene assays where its target-positive status had been confirmed. Milli-Q water was included as a no-template negative control to detect potential contamination. PCR products were separated by electrophoresis on 1.5% (w/v) agarose gels prepared in 1× TAE buffer. Following electrophoresis, DNA bands were visualized using a nucleic acid staining reagent and a gel documentation system. The presence or absence of specific amplification products was determined by comparison with a DNA molecular weight marker and the expected amplicon sizes. This targeted PCR analysis was restricted to the five selected genetic determinants and therefore did not provide a comprehensive assessment of all potentially transferable antibiotic-resistance genes or virulence-associated factors.

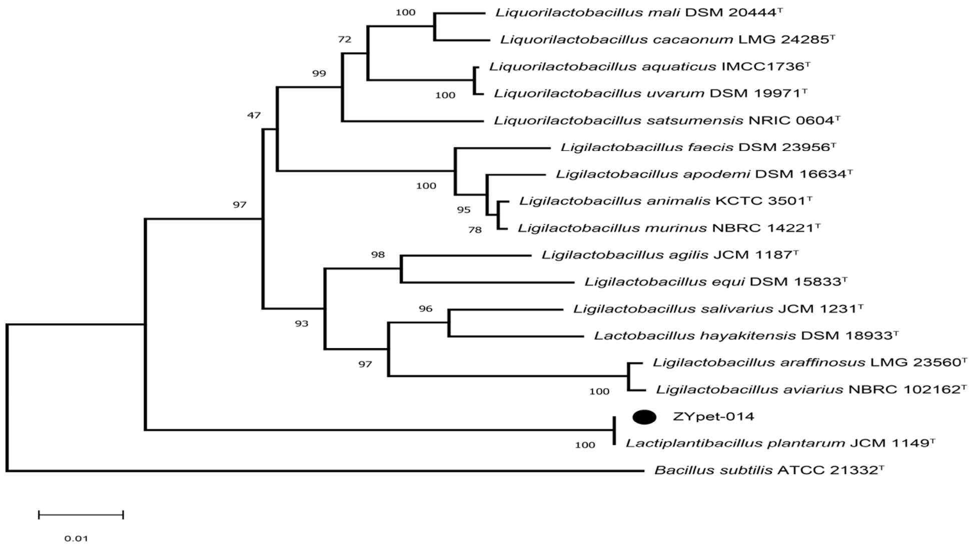

Supplementary Figure S2. Phylogenetic position of Lactiplantibacillus plantarum ZYpet-014 inferred from 16S rRNA gene sequences. ZYpet-014 clustered with L. plantarum JCM 1149^{T} with a bootstrap support value of 100. Bacillus subtilis ATCC 21332^{T} was included as the outgroup. Numbers at the nodes represent bootstrap support values, and the scale bar indicates 0.01 nucleotide substitutions per site.

Supplementary Table S1. Antibiotic susceptibility screening of Lactiplantibacillus plantarum ZYpet-014.

Isolate	GEN	CIP	CTR	E	AMP	TET	SXT	C	MY	PEN
ZYpet-014	I	I	I	S	S	I	S	S	I	S

Notes: S—susceptible; I—intermediate resistant; R—resistant. The concentrations of antibiotics are expressed in micrograms per disc (µg/disc): genmalicin (GEN, 10), ciproffoxacin (CIP, 5), ceftriaxone (CTR, 30), erythromycin (E, 15), ampicillin (AMP, 10), telracyclin (TET, 30), compound sulfamethoxa (SXT, 25), chloramphenicol (C, 30), lincomycin (MY, 2), and penicillin (PEN, 10).

Supplementary Table S2. Targeted screening of virulence-associated and antibiotic-resistance genes in ZYpet-014.

Isolates	Virulence Factors Genes			Antibiotic Resistance Genes	
	ace	gelE	cylA	vanA	tetM
ZYpet-014	-	-	-	-	-

Notes: -, the genes were not detected in the strains.
